# A new role for SR1 from *Bacillus subtilis*: regulation of sporulation by inhibition of *kinA* translation

**DOI:** 10.1093/nar/gkab747

**Published:** 2021-09-03

**Authors:** Inam Ul Haq, Sabine Brantl, Peter Müller

**Affiliations:** Matthias-Schleiden-Institut für Genetik, Bioinformatik und Molekulare Botanik, AG Bakteriengenetik, Friedrich-Schiller-Universität Jena, Philosophenweg 12, Jena D-07743, Germany; Matthias-Schleiden-Institut für Genetik, Bioinformatik und Molekulare Botanik, AG Bakteriengenetik, Friedrich-Schiller-Universität Jena, Philosophenweg 12, Jena D-07743, Germany; Matthias-Schleiden-Institut für Genetik, Bioinformatik und Molekulare Botanik, AG Bakteriengenetik, Friedrich-Schiller-Universität Jena, Philosophenweg 12, Jena D-07743, Germany

## Abstract

SR1 is a dual-function sRNA from *Bacillus subtilis*. It inhibits translation initiation of *ahrC* mRNA encoding the transcription activator of the arginine catabolic operons. Base-pairing is promoted by the RNA chaperone CsrA, which induces a slight structural change in the *ahrC* mRNA to facilitate SR1 binding. Additionally, SR1 encodes the small protein SR1P that interacts with glyceraldehyde-3P dehydrogenase A to promote binding to RNase J1 and enhancing J1 activity. Here, we describe a new target of SR1, *kinA* mRNA encoding the major histidine kinase of the sporulation phosphorelay. SR1 and *kinA* mRNA share 7 complementary regions. Base-pairing between SR1 and *kinA* mRNA decreases *kinA* translation without affecting *kinA* mRNA stability and represses transcription of the KinA/Spo0A downstream targets *spoIIE*, *spoIIGA* and *cotA*. The initial interaction between SR1 and *kinA* mRNA occurs 10 nt downstream of the *kinA* start codon and is decisive for inhibition. The *sr1* encoded peptide SR1P is dispensable for *kinA* regulation. Deletion of *sr1* accelerates sporulation resulting in low quality spores with reduced stress resistance and altered coat protein composition which can be compensated by *sr1* overexpression. Neither CsrA nor Hfq influence sporulation or spore properties.

## INTRODUCTION

Small regulatory RNAs (sRNAs) are the main posttranscriptional regulators in all three kingdoms of life and either act by base-pairing with their target RNAs and or by protein binding (rev. in 1). Over the past 20 years, a variety of approaches have been employed to discover chromosome-encoded sRNAs in a multitude of Gram-negative and Gram-positive species (rev. in 2,3). Whereas cis-encoded base-pairing sRNAs are completely complementary to their target RNAs and can form complete duplexes with them, trans-encoded sRNAs are only partially complementary to their—often multiple—target RNAs yielding only partial duplexes (rev. in 1). The majority of sRNAs have been found and intensively characterized in *Escherichia coli* and *Salmonella enterica* (rev. in 4) whereas only a few well-studied examples are known from Gram-positive bacteria, among them *Bacillus subtilis* (rev. in 5–8). Trans-encoded sRNAs can employ a variety of regulatory mechanisms, affecting either target RNA translation or stability. They can inhibit translation initiation by direct binding to the target RBS (ribosome binding site), blocking of a ribosome stand-by site or inducing structural changes around the RBS, activate translation, inhibit or promote target RNA degradation or induce premature transcription termination (rev. in 1,5,8). In some instances, translation inhibition and recruitment of an RNase for target RNA degradation are combined. Other mechanisms are target mRNA trapping by sponge RNAs (rev. in 1) or interference between sRNA-induced translation inhibition and Rho-dependent transcription termination (rev. in 9).

Since trans-encoded sRNAs comprise very short (6–10 nt) regions complementary to their target mRNAs, they often require RNA chaperones like Hfq or ProQ to facilitate target RNA binding by either increasing the binding rate or stabilizing the sRNA/target RNA complex (rev. in 1).

Hfq can, in addition, stabilize sRNAs, help releasing an inhibitory structure that sequesters the target RBS, recruit RNases to target mRNAs and promote or block Rho-dependent transcription termination (rev. in 10). The recently discovered ProQ is only encoded in the genomes of Gram-negative bacteria and seems to play a similar role as Hfq but at a different set of targets because it can—in contrast to Hfq—also bind structured RNAs (11). In contrast to Gram-negative bacteria, Hfq does not seem to play a general role in sRNA-mediated regulation in Gram-positives (rev. in 8) but alternative RNA chaperones might fulfill the role of Hfq or ProQ. One of them might be CsrA (see below).

The majority of sRNAs are untranslated, but a few of them comprise small ORFs and act, in addition to base-pairing to their complementary target mRNAs, as protein-coding mRNAs in the same or a different pathway (rev. in 12).

The 205 nt long SR1 from *Bacillus subtilis* is such a dual-function sRNA (12). It is transcribed from a σ^A^-dependent promoter under gluconeogenic conditions, whereas it is repressed under glycolytic conditions mainly by CcpN and to a minor extent by CcpA (13–16). Via 7 complementary regions, SR1 base-pairs with *ahrC* mRNA encoding the transcriptional activator of the arginine catabolic operons *rocABC* and *rocDEF* to inhibit its translation (17). Inhibition occurs by inducing structural changes around the *ahrC* RBS (18). In addition, SR1 codes for the small peptide SR1P (39 aa) that interacts with glyceraldehyde-3P-dehydrogenase GapA (19). This interaction increases binding of GapA to RNase J1 and significantly enhances RNase J1 activity (20–22). Both functions of SR1—the base-pairing and the peptide encoding—are remarkably conserved over one billion years of evolution (23). Whereas the RNA chaperone Hfq is required for *ahrC* translation (18), the RNA chaperone CsrA was recently discovered to slightly restructure *ahrC* mRNA to facilitate SR1 binding (24).

*Bacillus subtilis* forms endospores to survive nutrient starvation. Over the years, the complex regulatory network that governs sporulation and involves more than 500 of the 4200 *B. subtilis* genes (25) has been elucidated step by step (rev. in 26). Five histidine kinases, KinA, KinB, KinC, KinD and KinE, perceive and transmit environmental signals that finally result in phosphorylation of Spo0A, the central transcriptional regulator of the sporulation genes (rev. in 27). KinA is the major histidine kinase in the phosphorelay that regulates sporulation (28). Its threshold level governs the entry of *Bacillus subtilis* into sporulation (29).

**Figure 1.**
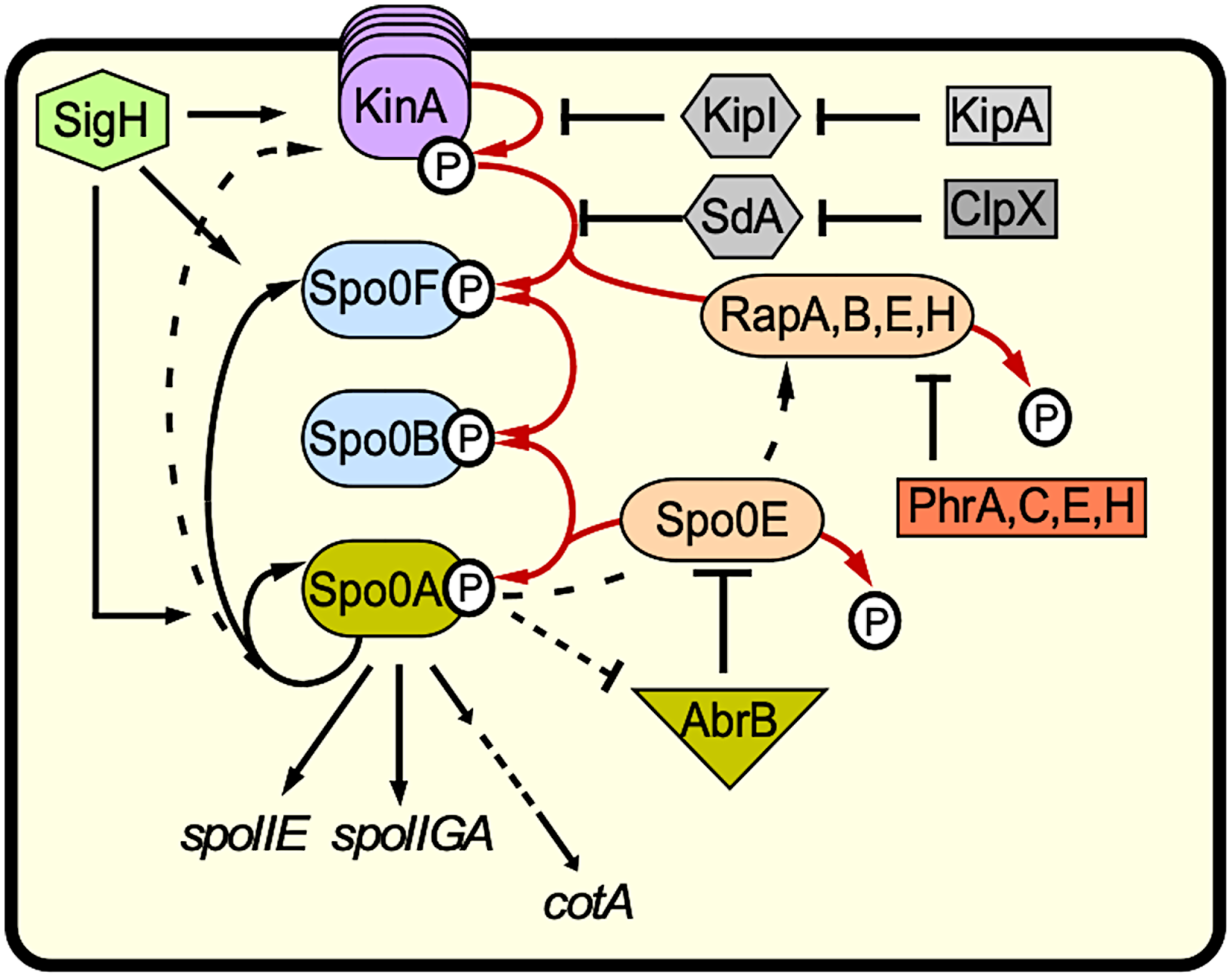
Role of KinA in sporulation. Central role of KinA in the sporulation cascade. KinA is the major histidine kinase in the sporulation phosphorelay whose transcription depends on stationary phase σ^H^. In addition, minor membrane-bound kinases KinB-E (stacked symbols behind KinA) transfer signals to Spo0A. KinA responds to an as yet unknown signal by autophosphorylating and subsequently transferring phosphate to Spo0F, which phosphorylates Spo0B, which passes the phosphate on to Spo0A, the master regulator of differentiation. At high levels, Spo0A∼P promotes sporulation by activating transcription of downstream genes, among them *spoIIE*, *spoIIGA* and, further downstream via SigF, SigE, SigG and SigK, to *cotA*. Several phosphatases (light orange) like RapA, B, E or H or Spo0E antagonize Spo0A phosphorylation at various steps. The Rap phosphatases are under control of the small phosphatase regulatory proteins (Phr) PhrA, C, E and H. By a direct protein-protein interaction with KinA, the small protein Sda blocks phosphate transfer to Spo0F, and is itself degraded by the ClpX protease. By contrast, KipI inhibits KinA autophosphorylation and is inhibited by KipA. In addition, transition state regulator AbrB represses transcription of the phosphatase *spo0E* gene and is itself repressed by Spo0A∼P.

Upon starvation and stress, KinA autophosphorylates and transfers its phosphate via Spo0F and Spo0B to Spo0A (rev. in 30, see Figure 1). KinA has three PAS domains that measure the redox status of the cell. Its transcription is under control of the stationary phase σ^H^. So far, only two direct regulators of KinA are known, the 46 aa protein Sda (suppressor of DnaA) and KipI (synonym PxpB) (Figure 1). Sda couples replication and sporulation and blocks the phosphate transfer to Spo0F during replication as its binding site on KinA overlaps with that of Spo0F (31). The Sda–KinA interaction surface was mapped (32). By contrast, KipI inhibits KinA autophosphorylation by affecting the ATP/ADP reactions but does not impact the phosphotransferase function of the KinA catalytical domain (33). Two KipI monomers bind via their C-terminal domains at a conserved proline in the KinA dimerization and histidine phophotransfer (DHp) domain (34). The KipI inhibitory activity is counteracted by KipA (33).

Here, we report on a new regulator of KinA, the dual-function sRNA SR1. We demonstrate that SR1 base-pairs with *kinA* mRNA upstream of the RBS and within the 5′ part of the coding sequence resulting in translation inhibition *in vivo* without altering the *kinA* mRNA stability. The *sr1*-encoded peptide SR1P is not involved in *kinA* regulation. Deletion or overexpression of *sr1* affect *B. subtilis* sporulation and transcription of at least three downstream KinA targets, the directly Spo0A-regulated genes *spoIIE* and *spoIIGA* as well as the σ^K^-dependent *cotA* gene which is located further down in the Spo0A regulatory cascade (see Figure 1). Furthermore, SR1 slows down the sporulation process and impacts spore size and hydrophobicity, stress resistance and coat protein composition. In contrast to its role in the SR1/*ahrC* system, neither Hfq nor CsrA affect sporulation. The physiological importance of sporulation control by SR1 is discussed.

## MATERIALS AND METHODS

### Enzymes and chemicals

Chemicals used were of the highest purity available. Q5 DNA polymerase, T7 RNA polymerase, CIP and polynucleotide kinase were purchased from NEB, Firepol Taq polymerase from Solis Biodyne, RNase T1 from Sigma Aldrich, RNase T2 from MoBiTec Göttingen, S1 nuclease from Thermo Scientific and RNase A and RNasin from Promega.

### Strains, media and growth conditions

*Escherichia coli* strain TG1 and *B. subtilis* strains DB104 (see Supplementary Table S2) were used. Complex TY medium (35) and CSE minimal medium (17) served as cultivation media. For *E. coli* 100 μg/ml ampicillin, 100 μg/ml spectinomycin and 200 μg/ml erythromycin were used and for *B. subtilis* 100 μg/ml spectinomycin, 5 μg/ml chloramphenicol, 5 μg/ml erythromycin and 12 μg/ml kanamycin.

### Sporulation assay, purification of endospores and spore coat proteins and phase-contrast microscopy

For sporulation assays, TY cultures inoculated from a fresh agar plate were grown for 6 h at 37°C, diluted in CSE medium with 0.05% glucose to OD_600_ = 0.2 and further incubated for 24 h in the shaker bath at 37°C. Dilutions were plated on TY plates to count total CFU (colony forming units). In parallel, cultures were incubated at 80°C for 20 min and dilutions plated on TY plates for spore counting. For calculations of the sporulation rate, the number of spores per total CFUs was considered.

Strains were cultivated for 48 h in CSE with 0.1% glucose (for nutrient starvation) or in TY (for nutrient rich conditions) by shaking at 37°C. Endospores were purified by repeated centrifugation, washing with ice cold bidist (bidistilled water) and storage at 4°C with repeated washing steps every 2–3 days for 4 weeks. One additional washing step was included before further use.

Spore coat proteins were prepared as described (36) with following modifications: Spores were treated for 30 min in lysozyme/TES buffer at 37°C followed by two washing steps in fresh TES buffer and 2 h incubation at 70°C in 100 mM sodium borate, 100 mM NaCl, 0.5% SDS and 50 mM DTT; pH 10.0. After centrifugation, the supernatant was denatured with Laemmli buffer and separated on a 12% Tris–glycine SDS-PAA (polyacrylamide gel).

For microscopy, purified spores were applied to an agarose pad (1% agarose in water) and phase contrast images obtained with a Nikon eclipse Ti2 microscope equipped with a Nikon Plan‐Apochromat 100×/1.45 oil immersion Ph3 objective and a Hamamatsu ORCA-Flash4.0 LT + Digital CMOS camera. For each sample, five pictures were taken from different spots. Spore length measurements were performed with ImageJ and manually for each spore, after the identity of the pictures was blinded. For each picture, the length and breadth of only 20 spatially singular spores were measured and spores of all rotary orientations were included. Halos around the spores were not included. Identities of the pictures were only revealed after evaluation of the measurements.

### Analysis of spore resistances and hydrophobicity

Purified spores were treated for 10 min in bidist at 70°C and subsequently subjected to different stress factors. To investigate heat resistance, spores were diluted in TY medium, incubated at 86°C, time samples taken and plated on TY plates, which were incubated overnight at 37°C for CFU counting. Survival rates were depicted as relative CFUs compared with a pre-treatment sample. For ethanol resistance, ethanol was added to a final concentration of 70% to spores kept in bidist followed by incubation at 65°C. Time samples were taken and diluted in fresh TY, plated, incubated and evaluated as above. To analyse spore hydrophobicity, white mineral oil was washed five times with equal amounts of bidist by vortexing and subsequent centrifugation to remove water-soluble components. Spores were diluted in bidist to 400 μl, of which 100 μl were plated on TY plates to determine total CFU. The rest was mixed with 300 μl washed mineral oil and vortexed for 20 s. After 10 min of spontaneous phase separation, 100 μl of the aqueous phase were plated on TY plates to determine the percentage of hydrophilic spores. Plates were incubated at 37°C overnight and CFU counted. Hydrophobicity was calculated as the difference between total spores and hydrophilic spores divided by the number of total spores.

### Quantification of AP (alkaline phosphatase) and DPA (dipicolinic acid)

Strains were grown for 8 h in 500 ml TY with 0.1% glucose at 37°C, centrifuged, resuspended in the same volume of CSE medium without glucose and further cultivated in a shaker bath. 1 ml samples were taken at different time-points, shock-frozen and stored at -20°C. AP activity was measured as described (37): Frozen samples were centrifuged, washed with and resuspended in 900 μl AP buffer (0.5 M Tris–HCl pH 10.0; 5 mM MgCl_2_; 1 mM ZnCl_2_). 100 μl of solution 1 (0.5% BCIP and 0.75% NBT in 25% DMF and 75% bidist) were added and the reaction stopped by adding 250 μl AP stop solution (1 M KH_2_PO_4_, 0.5 M EDTA pH 8.0). Samples were centrifuged, pellets washed three times with and resuspended in 500 μl bidist. Then, 500 μl DMF were added and cells were lysed for 1 h at 37°C. The optical density (OD_630_) of cleared lysates was measured against bidist/DMF. DPA quantification was performed as described (38) with slight modifications: 50 ml samples were centrifuged, washed twice with and resuspended in 1 ml bidist and stored at –20°C until further use. After 15 min treatment with 100 μl lysozyme (10 mg/ml) at 37°C, samples were autoclaved for 30 min at 121°C, subsequently acidified with acetic acid (0.5 M final concentration) and incubated at 37°C for 1 h. 800 μl of the cleared lysate were mixed with 200 μl 1% (NH_4_)_2_ Fe(SO_4_)_2_ and 1% ascorbic acid in bidist, and the optical density measured immediately at 440 nm.

### Primer extension

Primer extension was carried out as described (13) using total RNA from *B. subtilis* strain DB104 and 5'-labelled primers SB346 and SB3692 (all primers are listed in Supplementary Table S1). For the sequencing reaction, pUCSR1 (13) served as template.

### *In vitro* transcription, preparation of total RNA and Northern blotting

*In vitro* transcription was performed as described (18). Two-step PCRs were employed to introduce mutations by exchanging nucleotides of the complementary inner primers (Supplementary Table S1). Preparation of total RNA and Northern blotting including the determination of RNA half-lives were carried out as described (39).

### cDNA synthesis and qRT-PCR

RNA prepared from 500 μl stationary phase TY cultures was treated with 5 μl DNase I (10 U) in a total volume of 50 μl for 2 h at 37°C. After phenol and chloroform extraction followed by ethanol precipitation, cDNA was synthesized with 50 U SuperScript IV reverse transcriptase (Invitrogen) for 120 min at 42°C, followed by 10 min digestion with 1 μl RNase A (25 U) at 37°C. After one phenol and two chloroform extractions followed by ethanol precipitation, the pellet was dissolved in 100 μl bidist. Quantitative real-time PCR was performed with the Maxima SYBR Green/ROX qPCR Master Mix (ThermoFisher) and the Mx3005P system (Stratagene) in 96 well blocks. Each well contained 9 μl bidist, 1.5 μl of primer mix (5 pmol/ml each in bidist) and 2 μl undiluted template cDNA. The reaction was started after addition of 12.5 μl qPCR MasterMix 2× in a darkened room. Forty cycles with denaturation for 30 s at 95°C, 30 s annealing at 45°C and 30 s extension at 72°C were used. Only for 5S rRNA detection, the template cDNA was diluted 1000-fold. Immediately after qRT-PCR, a melting point analysis with MxPro software (Stratagene) was carried out to test for product specificity. For final validation, the ΔΔCt method was employed defining the number of cycles at which the fluorescence exceeds a certain threshold as Ct value. For evaluation, the MxProSoftware from Stratagene was used. Each experiment was performed with three independent clones per genotype and with a second technical replication of each sample.

### Analysis of RNA–RNA complex formation and secondary structure probing

Both SR1 and *kinA*_233_ mRNA were synthesized in vitro by T7 RNA polymerase from PCR templates. RNA-RNA complex formation assays were performed as previously (24). RNA secondary structure probing was conducted as described (40) with slight modifications: SR1 or *kinA* mRNA (20 000–30 000 cpm) were dissolved in 5 μl 1× TMN buffer containing 0.4 μg tRNA, the diluted RNases T1, T2, A or S1 nuclease were added and cleavage conducted for 5 min at 37°C. For S1 cleavage, 2 mM ZnCl_2_ were added. Reaction mixes were separated alongside a T1 ladder on denaturing 8% PAA gels. For mapping of the SR1-*kinA* mRNA complex, an excess of unlabelled RNA was used and complex formation allowed for 15 or 30 min in TMN buffer before addition of RNases.

### Construction of strains and plasmids and determination of β-galactosidase activities

To construct transcriptional *lacZ* fusions for Spo0A downstream targets, PCR fragments were generated on chromosomal DNA with primer pairs SB3545/SB3546 (*spoIIGA*), SB3543/SB3544 (*spoIIE*) and SB3547/SB3548 (*cotA*) each comprising –115 to + 10 relative to the transcription start site and including all known Spo0A binding sites, digested with BamHI and EcoRI and inserted into pMG16 (41) yielding pMGPGA(p*_spoIIGA_*), pMGPE(p*_spoIIE_*) and pMGPC(p*_cotA_*), respectively. The β-galactosidase activity was measured at 28°C as described (24), but transformants were inoculated from fresh agar plates into TY, grown for 2 h in the shaker bath at 37°C, subsequently diluted in prewarmed TY to OD_560_ = 0.2 and further cultivated for 24 h. Samples were taken after 4, 8, 16 and 24 h. To construct a translational *kinA-lacZ* fusion, a PCR fragment was generated on chromosomal DNA of strain DB104 with primer pair SB3501/SB3465, cleaved with BamHI and EcoRI and inserted into pGAB1 (24) yielding pGAB-kinA. The same approach was used to construct the pGAB-kinA_mD_, and primer pair SB3651/SB3652 was used to generate the mutation. Primer SB3501 adds the weak constitutive promoter pI (42) to the 5′ end of the *kinA* sequence composed of the native 5′ UTR and the first 100 codons. pGAB-kinA and pGAB-kinA_mD_ were linearized with ScaI and integrated into the *amyE* locus of the DB104 (*kinA::kan^R^*) strain. The β-galactosidase activities were determined at 55°C as above after 6 h growth at 37°C in a shaker bath. Plasmid pGKSR1_mD_S was constructed by cloning a BamHI/EcoRI fragment generated by a two-step-PCR with primer pairs SB350/SB3641 and SB3642/SB317 into the pGK15 BamHI/EcoRI vector. A list of all plasmids used is provided in Supplementary Table S3. DB104 containing a start-to stop codon mutation in the *sr1* gene was constructed by LFH (long-flanking homology)-PCR (39). A 1 kb front cassette with an *sr1* start-to stop mutation was generated by PCR with outer primer pair SB3771/SB3772 and internal primer pair SB3769/SB3770 on chromosomal DNA. A 1 kb back cassette was generated with primer pair SB3773/SB3774. The chloramphenicol resistance gene was amplified on plasmid PINT8C as template with primer pair SB2938/SB2939. The final 3 kb fragment generated by combining and amplifying the three cassettes with primer pair SB3772/SB3774 was directly used for DB104 transformation. All mutations were confirmed by sequencing.

## RESULTS

### CopraRNA predicts complementary base-pairing between SR1 and *kinA* mRNA

**Figure 2.**
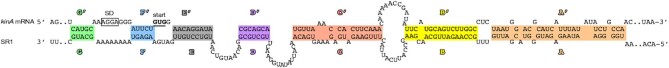
Complementarity between SR1 and *kinA* mRNA. IntaRNA (44) was used to search for complementary regions between SR1 and *kinA* mRNA. The seven mostly uninterrupted complementary regions are highlighted in colour and designated A to G in SR1 and A’ to G’ in *kinA* mRNA. The RBS is boxed and the GUG start codon is underlined.

So far, only one primary target of the sRNA SR1 is known, *ahrC* mRNA (17,18). Since the majority of base-pairing sRNAs from intergenic regions of bacterial chromosomes studied to date have several targets (1), CopraRNA (43) was employed to search for additional targets of SR1. Rank 1 target was *kinA* encoding the major kinase of the sporulation phosphorelay that phosphorylates Spo0F (rev. in 26). IntaRNA (44) predicted 7 more or less continuous regions in *kinA* complementary with SR1 which we designated A’ to G’ in *kinA* and A to G in SR1, among them two stretches, G’ and F’, flanking the RBS, respectively (Figure 2) and one, E’, directly downstream of the GUG start codon. The other regions were found between nt 70 and nt 154, i.e. within the first 32 codons of the *kinA* ORF.

### SR1 and *kinA* mRNA interact *in vitro*

**Figure 3.**
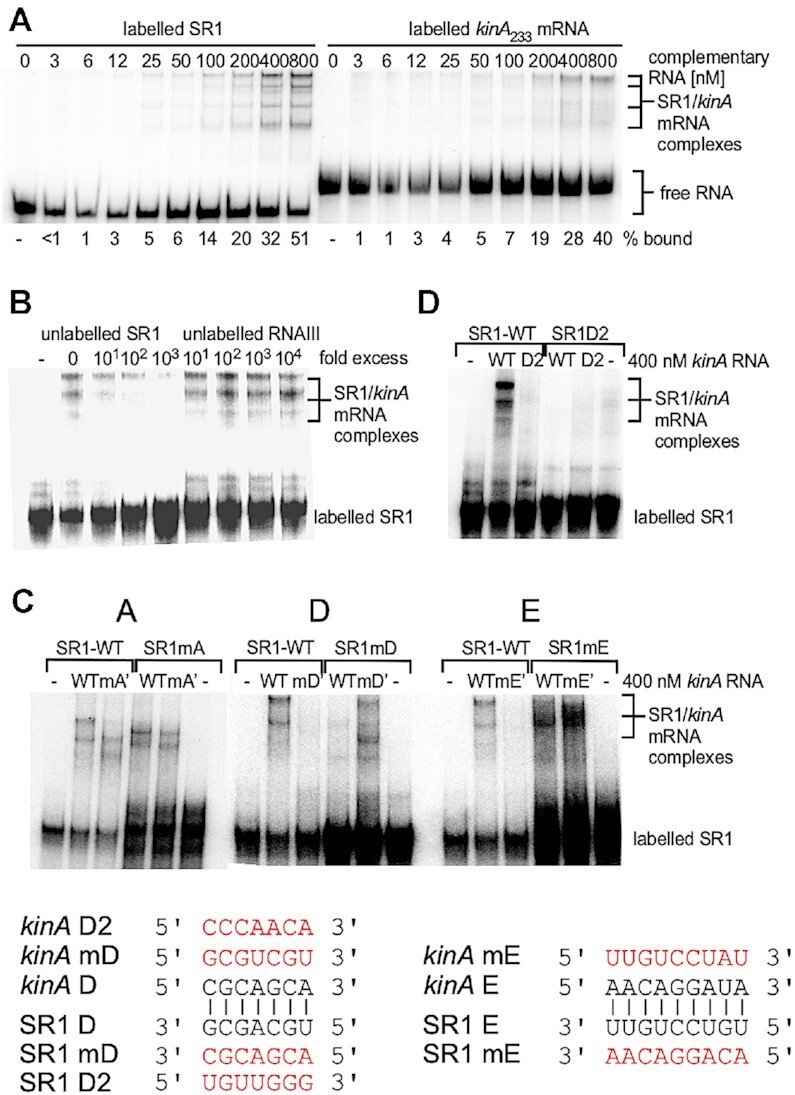
SR1 and *kinA* mRNA interact *in vitro*. EMSAs with 0.15 fmol ^32−^P [ α-UTP]-labelled RNA and increasing concentrations of unlabelled complementary RNA. Labelled and unlabelled RNA were mixed and incubated for 30 min at 37°C in TMN buffer, followed by separation on 6% native PAA gels. Autoradiograms of the gels are shown. (**A**) EMSAs with wild-type *kinA* mRNA and SR1 species. (**B**) Competition EMSA with unlabelled SR1 or heterologous RNAIII. Above, the fold excess of the competitor RNA is indicated. (**C**) EMSAs with SR1 and *kinA_233_* mRNA species either wild-type or mutated in region A or A’, D or D’ or E or E’. In the mutated RNA (labelled with m) all nt of the corresponding region were exchanged by the respective complementary nt as shown below for *kinA*mD and *kinA*mE. (**D**) as (C), but only 2 nt in region D or D’ were exchanged (see below *kinA*D2, SR1D2)

To investigate whether SR1 is able to base-pair with *kinA* mRNA, we used labelled wild-type SR1 and an excess of unlabelled *kinA* mRNA (comprising the 5′ 233 nt) and vice versa in EMSAs. Up to four SR1-*kinA* RNA complexes most likely representing different conformations of 1:1 complexes, could be observed (Figure 3A). 51% of labelled SR1 were found in the complex with *kinA* mRNA at a concentration of 800 nM (Figure 3A). A time course experiment with labelled RNA and 400 nM of the unlabelled binding partner demonstrated an increase of complex formation from 1 min towards 30 min (Supplementary Figure S1). To confirm that binding is specific to SR1, a competition experiment was performed by adding an excess of unlabelled SR1 or unlabelled heterologous RNAIII (42) to labelled SR1 and an excess of unlabelled *kinA* mRNA (Figure 3B). While a 100-fold excess of unlabelled SR1 could successfully displace labelled SR1 from the complex with *kinA* mRNA, unlabelled RNAIII was not even able to do so at a 10^4^-fold excess. To narrow down complementary regions decisive for the SR1-*kinA* RNA interaction, each of the 7 complementary regions A to G were mutated individually in either *kinA* mRNA or SR1 (Figure 3C and Supplementary Figure S1B). In each mutant, all nt of the corresponding region (see coloured regions in Figure 2) were replaced by the complementary nt. Interestingly, only mutated regions D and E of *kinA* prevented the interaction with SR1 while mutations in A, B, C, F and G did not impair binding (Figure 3C and Supplementary Figure S1B). Region D located 10 nt downstream of the *kinA* start codon was decisive for SR1 binding, which was confirmed by compensatory mutations (Figure 3C). Even the alteration of 2 nt within region D prevented SR1 binding to *kinA* mRNA and vice versa, but in contrast to an exchange of 7 nt they could not completely compensate the binding deficiency of the individually mutated region (Figure 3D).

In summary, SR1 binds specifically to *kinA* mRNA *in vitro*, and complementary region D located 10 nt downstream of the *kinA* start codon is decisive for the *in vitro* SR1-*kinA* RNA interaction.

### SR1 represses *kinA* mRNA translation *in vivo* by a base-pairing interaction but does not affect *kinA* mRNA stability

**Figure 4.**
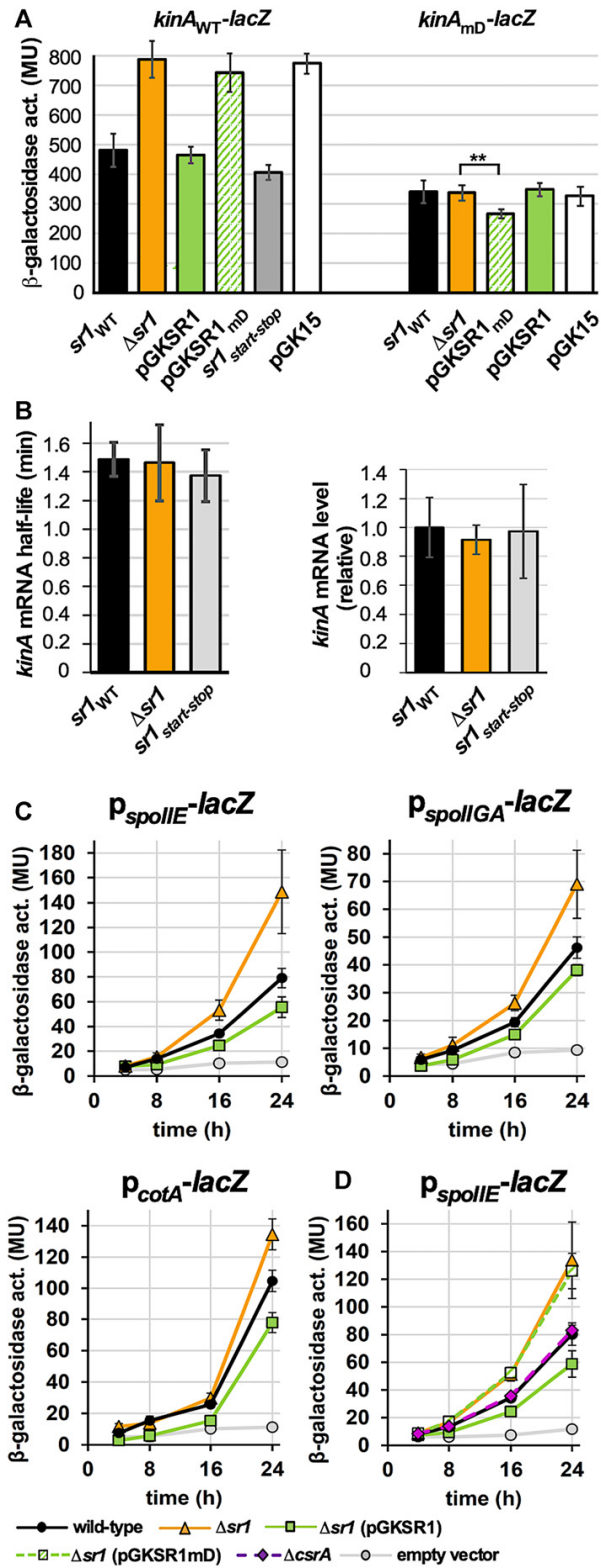
SR1 inhibits *kinA* translation without impacting RNA stability and affects transcription from three Spo0A∼P-dependent promoters. (**A**) Left: A translational *kinA*-*lacZ* fusion under the weak constitutive heterologous promoter pI were integrated into the *amyE* locus of *B. subtilis* DB104 *(*Δ*kinA*) and the isogenic Δ*sr1*, *sr1_start-stop_*, Δ*sr1* (pGKSR1S), Δ*sr1* (pGKSR1_mD_S) and Δ*sr1* (pGK15) strains. Right: A translational *kinA_mD_*-*lacZ* fusion under control of pI was integrated into the DB104 *(*Δ*kinA*) strain and the isogenic Δ*sr1*, Δ*sr1* (pGKSR1mD) and Δ*sr1* (pGKSR15) strains. KinA_mD_ and SR1_mD_ are complementary. The β-galactosidase activities were measured from six to eight individual clones (*kinA_WT_-lacZ*) or at least 10 individual clones (*kinA_mD_-lacZ*) for each strain after 6 h growth in TY medium until OD_600_ = 4.5. The indicated values are the results of three biological replicates. Error bars represent standard deviations. pGK15, empty vector control; asterisks label significancy in Student's *t*-test (** *P* < 0,005). (**B**) Left: The half-life of *kinA* mRNA was determined by qRT-PCR as described in *Materials and Methods* in wild-type DB104 and the isogenic *(*Δ*sr1*) and *sr1_start-stop_* strains. The average values obtained from three biological replicates with standard deviations are shown. Right: The relative amounts of *kinA* mRNA were determined by qRT-PCR in the wild-type strain DB104 and the isogenic *(*Δ*sr1*) and *sr1_start-stop_* strains after growth in complex TY medium until OD_600_= 4.5. (**C**) Transcriptional p*_spoIIE_*-*lacZ*, p*_spoIIGA_*-*lacZ* and p*_cotA_*-*lacZ* fusions were integrated into the *amyE* locus of *B. subtilis* strain DB104, DB104*(*Δ*sr1*) and DB104*(*Δ*sr1*) (pGKSR1) and β-galactosidase activities measured after growth in TY medium after 4, 8, 16 and 24 h. The indicated values are the results of three biological replicates with seven transformants each. Error bars represent standard deviations. EV: empty vector control (insert-free pMG16 vector integrated in *amyE* locus). (**D**) Comparison of the effect of pGKSR1 and pGKSR1*_mD_*as well as CsrA on the transcription from p*_spoIIE_*.

To confirm the *in vitro* interaction between SR1 and *kinA* mRNA *in vivo*, a translational *kinA-lacZ* reporter gene fusion under control of the weak constitutive heterologous promoter pI (42) was constructed and integrated into the *amyE* locus of the *B. subtilis* chromosome of the Δ*kinA* and the isogenic Δ*sr1* strain as well as the *sr1* overexpression strain Δ*sr1* (pGKSR1S). As shown in Figure 4A left, deletion of *sr1* resulted in a 1.8-fold increase in *kinA* translation whereas *sr1* overexpression was able to compensate this effect. This suggests that SR1 acts on *kinA* posttranscriptionally. To investigate if the *sr1* encoded small protein SR1P is involved in the regulation of *kinA*, an isogenic *B. subtilis* strain with a start-to stop codon mutation in the chromosomal *sr1p* ORF was constructed. This mutation had no impact on the *kinA*-*lacZ* translation indicating that only the sRNA SR1 but not SR1P was required for the observed effect (Figure 4A). To analyse a possible influence of SR1 on the *kinA* mRNA stability, we employed qRT-PCR to determine the half-life of *kinA* mRNA in the presence and absence of SR1 as well as in the absence of the *sr1-*encoded peptide SR1P. As shown in Figure 4B left, neither SR1 nor SR1P affected the half-life of *kinA* mRNA. In addition, the qRT-PCR indicated that the amounts of *kinA* mRNA were almost the same in the wild-type, *sr1* knockout and *sr1_start-to stop_* strain (Figure 4B, right) ruling out an effect of SR1 on the *kinA* promoter. Within the 5′ 233 nt of *kinA* mRNA we did neither find a potential for alternative folding nor a Rho-dependent or Rho-independent transcription terminator. Furthermore, a previous *B. subtilis* transcriptome analysis performed in the absence and presence of Rho (45) did not reveal any premature termination signal in the presence of Rho in the corresponding region. Therefore, we can exclude SR1-mediated transcription termination as alternative mechanism of SR1 action. Consequently, SR1 represses translation of *kinA* mRNA without affecting its stability.

To substantiate the importance of complementary region D for the SR1-*kinA* mRNA interaction *in vivo*, we constructed pGKSR1_mD_S carrying the same 7 nt exchange in region D which was shown in the EMSAs (Figure 3C) to be important for *kinA* mRNA binding. This mutation does not affect the previously demonstrated ability of SR1P to interact with GapA for modulating the *B. subtilis* degradosome-like network (20,21). The β-galactosidase activities in the Δ*sr1* strain with and without pGKSR1_mD_S were comparable (Figure 4A), indicating that SR1_mD_ cannot downregulate translation of wild-type *kinA* mRNA. Northern blots confirmed identical expression levels of SR1, SR1_start-to stop_ and SR1_mD_ (Supplementary Figure S2).

To confirm a base-pairing interaction between SR1 and *kinA* mRNA *in vivo*, compensatory nt exchanges were introduced in SR1 and *kinA* in the translational *kinA-lacZ* fusion and β-galactosidase activities measured (Figure 4A right). The β-galactosidase activities of the *kinA_mD_-lacZ* fusion were almost the same in the presence and absence of wild-type SR1 demonstrating that wild-type SR1 cannot interact with *kinA_mD_* mRNA. By contrast, SR1_mD_ expressed from pGKSR1_mD_ was able to interact with the *kinA_mD_* mRNA indicated by a slightly, but significantly, lower β-galactosidase activity of the *kinA_mD_-lacZ* fusion.

From these data, we conclude that the base-pairing interaction between SR1 and *kinA* mRNA is decisive for the repression of *kinA* translation *in vivo*.

### Analysis of three promoters regulated by the KinA-downstream target Spo0A corroborates the *in vivo* role of SR1 in sporulation

To investigate the impact of SR1 on the transcription of KinA downstream genes, a set of three genes was chosen whose transcription is strongly induced by Spo0A∼P during sporulation (45,46). While *spoIIE* and *spoIIGA* are σ^F^-dependent early sporulation genes encoding SpoIIE and SpoIIGA, respectively, *cotA* is a σ^K^-dependent late sporulation gene. SpoIIE is a serine phosphatase that dephosphorylates the anti-anti-sigma factor SpoAA and controls σ^F^ activity whereas SpoIIGA is a protease involved in the maturation of σ^E^. CotA is an outer spore coat protein (47). Transcriptional fusions of the p*_spoIIE_*, p*_spoIIGA_* and p*_cotA_* promoters with the promotorless *lacZ* gene were constructed and integrated into the chromosomal *amyE* locus of *B. subtilis* strains DB104 and isogenic Δ*sr1* and Δ*sr1*(pGKSR1) strains. Seven independent transformants were grown over 24 h in complex TY medium, time samples taken and β-galactosidase activities determined (Figure 4D). In all three cases, deletion of *sr1* increased the promoter activity, whereas *sr1* overexpression under its native promoter from plasmid pGKSR1 (15 copies/cell) decreased the promoter activity. The most pronounced effects were observed for the *spoIIE* promoter. After 24 h growth (conditions of sporulation) an almost 2-fold increase in p*_spoIIE_* activity was observed in the absence of SR1, whereas the effects for p*_spoIIGA_* and p*_cotA_* were only 1.5- and 1.3-fold, respectively. The reduction of promoter activity upon *sr1* overexpression was generally much smaller, but over the time—after 8, 16 and 24 h—the corresponding values were always lower than those of the wild-type strain. Furthermore, the p*_spoIIE_* activity was compared between the Δ*sr1* strain without and with pGKSR1_mD_S and found to be nearly identical (Figure 4C) supporting the importance of region D for the SR1-mediated inhibition of *kinA*.

From these data, we conclude that the regulation of the Spo0A downstream genes affected by the major kinase KinA is due to the base-pairing between SR1 and *kinA* mRNA.

### Secondary structure probing of *kinA* mRNA and the SR1-*kinA* mRNA complex

To investigate in more detail how SR1 can inhibit *kinA* translation without directly binding to and protecting the *kinA* RBS, we first determined the secondary structure of the 233 nt *kinA* mRNA species that was also used in the EMSAs. To this end, we performed limited digestions with structure-specific ribonucleases by treating *in vitro* synthesized, 5′-end labelled and gel-purified *kinA_233_* mRNA with RNase T1 (cleaves 3′ of unpaired G residues), RNase T2 (unpaired nucleotides with a slight preference for A residues), RNase A (unpaired C and U residues) and nuclease S1 (single-stranded nucleotides). Supplementary Figure S3A shows the analysis and Supplementary Figure S3B the schematic presentation of two slightly different *kinA* mRNA structures derived from the cleavage data, one 5 bp stem below regions G’ to D’ the other with an 8 bp stem interrupted by a bulged out U. Most probably, a mix between both structures is present *in vitro*. However, in both structures, the double- or single-strandedness of all complementary regions is identical.

Structure probing revealed that the *kinA* RBS (nt 46–49) is located in a single-stranded region (nt 44 to 52) and should, therefore, be accessible to the ribosomal 30S subunit. Interestingly, the complementary region G’ upstream of the RBS as well as regions F’ and E’ immediately downstream of the RBS are located in completely (G’, F’) or almost completely (E’) double-stranded regions. Region D’ that proved to be decisive in the EMSA for interaction with SR1 (Figure 3C,D) displayed 4 single-stranded and 3 double-stranded nt whose complete (7nt) exchange lead DB104 (Δ*sr1*, pGKSR1_mD_) behave like the isogenic Δ*sr1* strain (Figure 4D). Region A’ was fully and C’ partially single-stranded whereas region B’ was completely paired.

**Figure 5.**
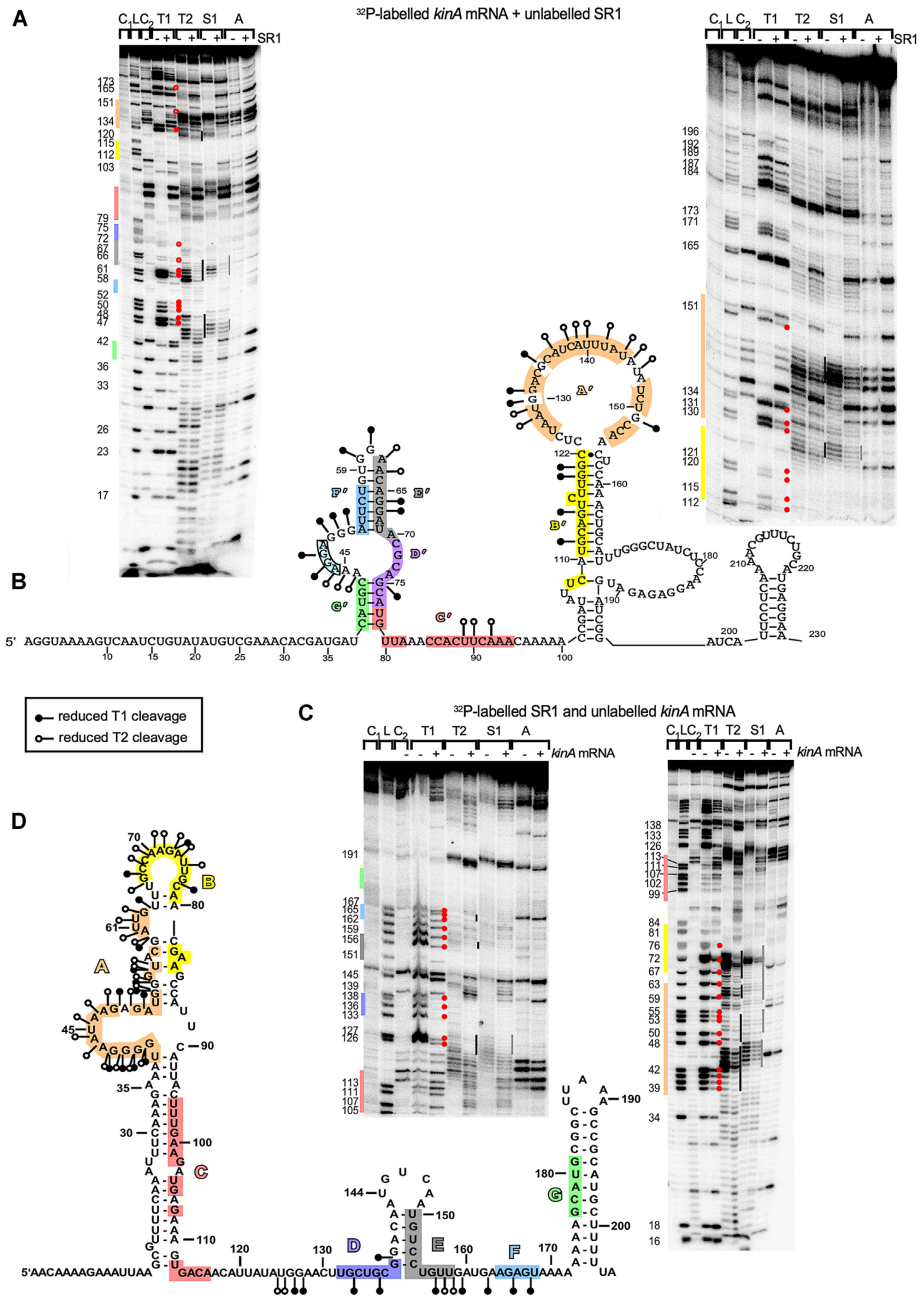
Secondary structure of the *kinA* mRNA/SR1 complex. (**A** and **C**) Secondary structure probing of the *kinA* mRNA/SR1 complex. 15 nmol of purified, 5′ labelled *kinA* mRNA or 5′ labelled SR1 were incubated with a 166-fold or 133-fold, respectively, excess of the complementary unlabelled RNA, the complex allowed to form for 30 min at 37°C and subjected to limited cleavage of T1 (0.1 U), T2 (0.1 U), S1 (2.2 U) and A (0.4 ng). The digested RNAs were separated on 8% denaturing gels. Autoradiograms are shown. L, T1 digestion under denaturing conditions. Nucleotide positions are included. Altered T1 and T2 cleavages are indicated by the symbols shown in the box in (B) and (D). (**B** and **D**) Structures of *kinA* mRNA and SR1, respectively, in which nucleotides protected by binding of the complementary RNA are indicated.

The same approach was used for secondary structure probing of 5′-end labelled SR1, and the result confirmed our previously published SR1 structures (18,24): SR1 regions A, B, D, E and F were mostly or fully (region F) single-stranded and regions C and G were completely base-paired (Figure 5D).

To investigate protection of *kinA* mRNA by SR1 and the possible induction of structural alterations, 5′ labelled *kinA_233_* mRNA was incubated with an excess of unlabelled SR1, the complex was allowed to form at 37°C and, subsequently, partially digested with RNases T1, T2, A and nuclease S1 (Figure 5A, B). In parallel, 5′ end-labelled SR1 was incubated with an excess of unlabelled *kinA_233_* mRNA and treated likewise (Figure 5C, D).

Addition of unlabelled SR1 to labelled *kinA* mRNA resulted in a clear protection of regions A’, B’, C’, D’ and E’ (Figure 5A), whereas regions G’ and F’ were already double-stranded in unbound *kinA* mRNA and could not be evaluated. Most important, however, was the complete protection of nt 44–52 covering the *kinA* RBS which itself is not complementary to any region in SR1. Apparently, SR1 binding to the adjacent complementary regions G’ and F’, E’ and D’ impedes the access of the 30S subunit to its binding site. This is in good agreement with the observed inhibition of *kinA* translation by SR1 (Figure 4A). These data were complemented by addition of unlabelled *kinA* mRNA to labelled SR1, which revealed protection of regions A, B, D, E and F (Figure 5C). Regions C and G could not be assessed, as they remained sequestered by intramolecular base-pairing.

In summary, secondary structure probing of the SR1-*kinA* mRNA complex demonstrated that SR1 binding makes the *kinA* RBS inaccessible, which is consistent with SR1 inhibiting translation of a *kinA-lacZ* fusion.

### Binding pathway

**Figure 6.**
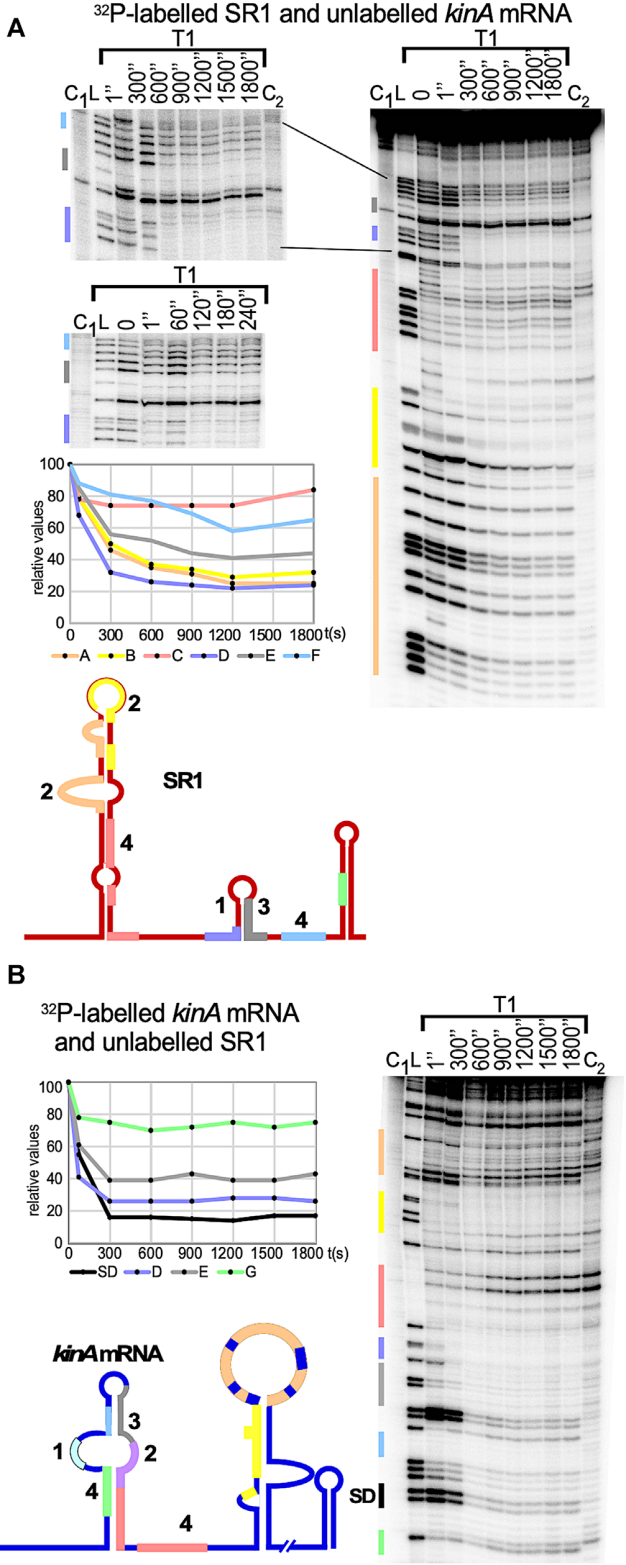
Time-course of SR1–*kinA* RNA interaction. The same approach was used as in Figure 5 for secondary structure probing with either labelled SR1 or labelled *kinA* mRNA. However, an excess of the complementary unlabelled RNA was added, incubation started at 37°C and samples taken at the indicated incubation times, instantly added to tubes containing RNase T1 and subjected to a 5 min digestion at 37°C. (**A**) Time course with labelled SR1. The second smaller gel shows samples taken at shorter time intervals and was used to confirm that region D is bound first. (**B**) Time course with labelled *kinA* mRNA. In both cases, graphs based on the calculation of the corresponding gels show that region D is bound first, whereas regions G and C, that are completely double-stranded in SR1 (G and C) and partially double-stranded in *kinA* mRNA (G’), do not play a role. In *kinA* mRNA, immediately after binding of D’, the RBS is protected. Numbers added to the regions in the schematic structures of SR1 and *kinA* mRNA indicate the sequence of interactions.

The results of the EMSAs (Figure 3) and the translational *lacZ*-fusions (Figure 4A) revealed that region D/D’ is decisive for the SR1-*kinA* mRNA interaction. To analyse the sequence of interactions between SR1 and *kinA* mRNA, two time-course experiments were performed, one with labelled SR1 and unlabelled *kinA* mRNA (Figure 6A), the other *vice versa* (Figure 6B). In both cases, the interaction between D and D’ was the initial one. It occurred already after a few seconds as seen in the high-resolution time course in Figure 6A. In *kinA* mRNA, the next protected region was the RBS, although it is not contacted directly by SR1. As more G-residues sensitive to RNase T1 cleavage are present in the complementary regions A to G in SR1 compared to A’ to G’ in *kinA* mRNA, T1-cleavages revealed that both the bulge in A and the loop of B bind more or less simultaneously after D, followed by the E and then the F region (Figure 6A). In the EMSAs (Figure 3), E was the only other complementary region whose mutation in SR1 prevented *kinA* mRNA binding whereas this was not the case in regions A, B, C, F or G. In *kinA*, region E bound immediately after D (Figure 6B) and, since B and F are completely double-stranded, no conclusion could be drawn about them.

In conclusion, the SR1–*kinA* mRNA interaction commences between complementary regions D and D’ located 10 nt downstream of the *kinA* start codon and leads to an immediate protection of the *kinA* RBS.

### SR1 affects *B. subtilis* sporulation

**Figure 7.**
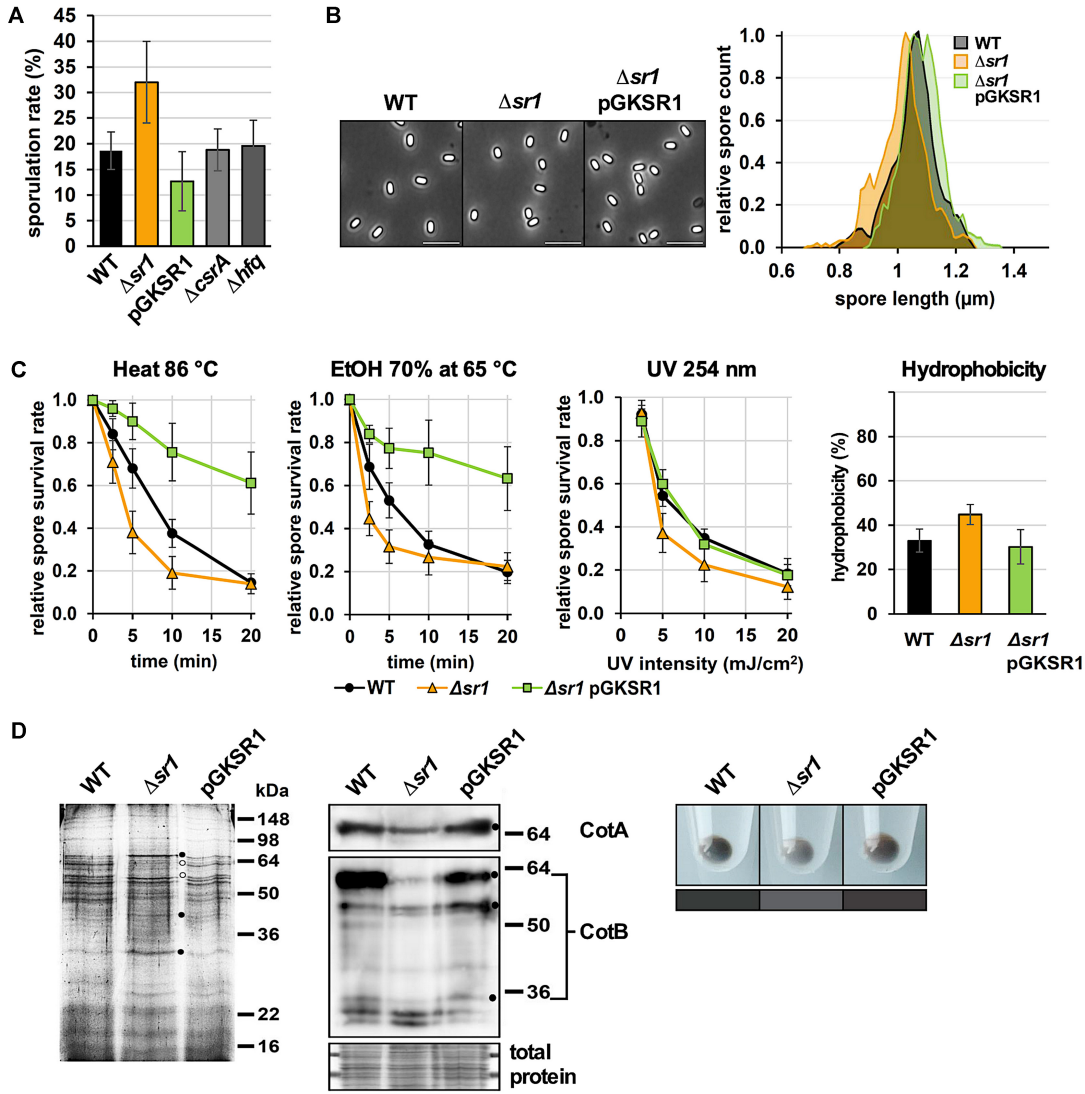
SR1 impacts *B. subtilis* sporulation, size and stress resistance of spores and abundance of spore coat proteins. (**A**) The sporulation assay was performed as described in Materials and Methods with strains DB104, DB104*(*Δ*sr1::cat*), DB104(pGKSR1), DB104*(*Δ*csrA::cat*) and DB104*(*Δ*hfq::cat*). In all cases, the indicated values are the results of three biological replicates. Error bars represent standard deviations. (**B**) Left: Phase-contrast micrographs of purified wild-type, *Δsr1* and *Δsr1* pGKSR1 spores. Right: Spore length distribution of wild-type (black), *Δsr1* (orange) and *Δsr1* pGKSR1 (green) spores. The lengths of 500 spores for each phenotype were measured from phase-contrast micrographs as shown left. Depicted are the smoothened histograms of the length distributions. (**C**) Resistance of spores obtained from DB104, DB104*(*Δ*sr1*) and DB104*(*Δ*sr1*, pGKSR1) against heat, ethanol, UV light and their hydrophobicity were determined in three independent experiments. Standard deviations are indicated. (**D**) Composition of spore coat proteins from wild-type, *Δsr1* and *Δsr1* pGKSR1 strains. Left, Coomassie-blue stained protein gel. Dots depict differences in the abundance of five individual proteins. Centre, Western blot with antibodies against CotA and CotB. CotB-46 is the main species, and CotB-66 likely a polyphosphorylated form of the protein (67). Below, the same amounts of total proteins applied for Western blotting were stained with Coomassie-blue as loading control. Right, spore colours. Below, the mean colours of comparable areas of the picture are represented for better comparison.

The translational repression of *kinA* by SR1 should impact *B. subtilis* sporulation. Therefore, we compared sporulation of wild-type strain DB104, DB104*(*Δ*sr1*) and the *sr1* overexpression strain DB104(pGKSR1). Cells were grown for 6 h in TY medium, inoculated to OD_600_ = 0.2 into minimal CSE medium with glucose and further cultivated in a shaker bath for 24 h. Afterwards, the ratio of spores and living cells was determined (Figure 7A). As expected, deletion of SR1 increased sporulation about 1.8-fold, whereas *sr1* overexpression from its native promoter reduced sporulation about 1.5-fold. Since CsrA binds SR1 (24), we investigated a potential influence of CsrA on sporulation. However, deletion of *csrA* had no significant effect on sporulation. Likewise, the deletion of *hfq* - Hfq also binds to SR1 (18) - did not affect sporulation (Figure 7A).

### The amount of SR1 affects properties, stress resistance and protein composition of spores

First, we performed an experiment to analyse if the amount of SR1 affects spore germination. To this end, purified spores from wild-type DB104 and the isogenic *sr1* knockout and pGKSR1 overexpression strains were treated for 10 min at 70°C, suspended in TY and cultivated at 37°C in a shaker bath. Aliquots were plated on TY agar plates at particular time points, incubated at 37°C overnight and CFUs were counted. All three strains showed a lag phase of about 190 min indicating that none of them displayed a germination latency (Supplementary Figure S4). Moreover, no differences in spore outgrowth ratio or kinetics between these strains were observed after germination induction with TY medium in time-lapse microscopy (not shown).

To investigate a possible effect of SR1 on spore size, phase-contrast microscopy was applied and the length of 500 spores measured for the wild-type, *sr1* knockout and the *sr1* overexpression strains. Spores from the Δ*sr1* strain were on average ≈ 5% shorter and those from the overexpression strain slightly longer than those from the wild-type strain (Figure 7B).

In addition, we compared purified spores for their outgrowth ability after stress treatment. Spores were treated for 10 min at 70°C, suspended in TY medium, incubated at 86°C and time samples plated on TY agar plates. After overnight incubation at 37°C, CFUs were counted. Figure 7C reveals that spores from the *sr1* overexpression strain showed a much higher heat resistance than those from the wild-type strain DB104 over the entire time, whereas the spores of the *sr1* knockout strain were less heat-resistant and formed less colonies, which was detectable already after 2.5 min of heat treatment. To evaluate another stress factor, spores were treated with 70% ethanol at 65°C and germination investigated as above. A similar effect was observed as for exclusive heat treatment (Figure 7C). Neither 65°C nor 70% ethanol alone had an appreciable effect on spore survival, even after a 60 min treatment (not shown). Interestingly, in both cases the survival rate of the Δ*sr1* strain caught up with the wild-type strain after ≈20 min. This could be an evidence that just a subpopulation of the Δ*sr1* spores had a reduced resistance. A similar result was observed after treatment with UV light (254 nm). Again, the Δ*sr1* strain was less resistant compared to wild-type and overexpression strains but neither the Δ*sr1* strain caught up with the wild-type strain with increasing UV stress nor was the *sr1* overexpression strain able to further increase its UV resistance above wild-type level. Furthermore, the hydrophobicity of Δ*sr1* spores was about 30% higher than that of the other two strains (Figure 7C, right).

To analyse a possible influence of SR1 on the protein composition of the spore coat, spore coat proteins from the three isogenic strains were prepared and analysed in a 12% SDS-PAA gel (Figure 7D). The abundance of at least five proteins differed between the three strains (labelled with dots). As shown by Western blotting, the amount of CotA and CotB was lower in spores formed in the absence of SR1 (Figure 7D) which might explain their lower stress resistance. In addition, we noticed a less brownish colour of Δ*sr1* spores.

Taken together we conclude that SR1 has a physiological function in spore formation, stress resistance and protein composition of the spore coat by regulating *kinA* translation. Furthermore, we hypothesize that under starvation conditions SR1 might slow down spore formation to enable the formation of high-quality spores.

### SR1 decelerates spore formation

**Figure 8.**
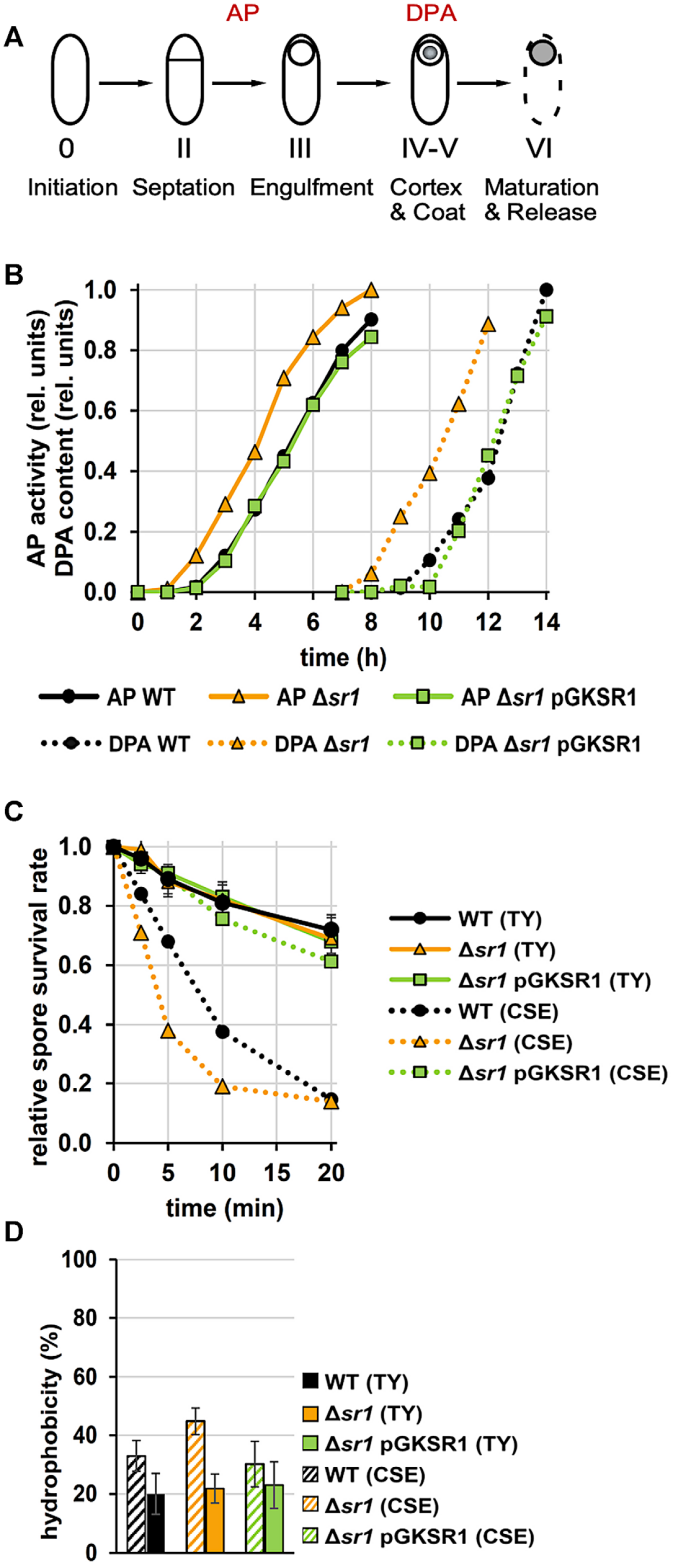
SR1 slows down sporulation and is only required under starvation conditions for production of high-quality spores. (**A**) Schematic representation of the sporulation process. Sporulation-specific alkaline phosphatase (AP) activity emerges between stages II and III. DPA is synthesized during stage V. (**B**) Investigation of the spore formation progress in wild-type, *sr1* knockout and *sr1* overexpression strains. AP activity and DPA content were measured over time after resuspension in CSE medium and are depicted as relative signal strength. The time difference between onset of AP activity and DPA detectability is representative of the sporulation progress. (**C**) Heat resistance assay of spores formed in TY medium. Purified spores of wild-type, *sr1* knockout and *sr1* overexpression strains cultured in TY were treated with 86°C and spore survival was measured over time. For comparison, the heat resistance of spores from minimal CSE medium (as in Figure 5C) is depicted in dotted lines. (**D**) Comparison of spore hydrophobicity between TY and CSE medium.

To investigate our hypothesis, we studied the impact of SR1 on the speed of spore formation. To this end, we chose two markers, AP (alkaline phosphatase) which is active between septation (stage II) and engulfment (stage III), and DPA (dipicolinic acid) detectable only after cortex and coat formation in stages IV and V of the spore formation process (Figure 8A). We grew the isogenic wild-type, Δ*sr1* and *sr1* overexpression strains and determined the sporulation specific AP activity and the presence of DPA during sporulation. As shown in Figure 8B, the AP activity could be measured ≈1 h earlier in the *sr1* knockout compared to the wild-type and *sr1* overexpression strains indicating that engulfment starts earlier in the absence of SR1. Likewise, DPA could be detected in the Δ*sr1* strain ≈ 2h earlier than in the wild-type and the *sr1* overexpression strain. This confirms our hypothesis that the *sr1* knockout strain sporulates earlier and progresses faster through spore formation than the wild-type strain. From these data, we infer that SR1 slows down the entire process of spore formation by about 2-fold repressing *kinA* translation and, consequently, reducing the amount of the major histidine kinase KinA.

### SR1 is only required under nutrient starvation in minimal medium for high quality spores

As SR1 is upregulated in the absence of glucose (13,15), we compared the properties of spores generated in minimal CSE with those generated in complex TY medium. In TY, SR1 is expressed in high amounts after 5 h (13), but the secondary carbon sources of the complex medium prevent the culture from starving. As presented in Figure 8C, spores generated in TY medium were more heat resistant in comparison to those generated in CSE, and heat resistance did not differ between wild-type, *sr1* knockout and *sr1* overexpression strains. By contrast, spores generated in CSE medium were less heat resistant and displayed differences between the three strains: Whereas spores from the *sr1* overexpression strain generated in CSE were only slightly less resistant than all spores generated in TY medium, spores from the wild-type strain showed a 2- to 3-fold lower heat resistance and those from the *sr1* knockout strain were even less heat resistant than those from the wild-type strain, at least after 5–15 min heat treatment. In addition, spores produced in TY were also less hydrophobic indicating a more efficient crust formation and general condition, and did not reveal differences between the three strains (Figure 8D). Together this illustrates that SR1 is only necessary under starvation conditions to enable the formation of high-quality spores.

### A sporulation-dependent promoter upstream of p_sr1_ barely contributes to the amounts of SR1

Previously, we mapped the *sr1* transcription start site (TSS) at a σ^A^-dependent promoter (13). Nicolas *et al.* reported an additional sporulation-dependent RNA originating ≈130 nt upstream of this TSS (45, see Supplementary Figure S5). Primer extension with cultures grown in TY for 6 h (stationary phase) or 24 h (sporulation) revealed the TSS of σ^A^-dependent p*_sr1_* after 30 min exposure (Supplementary Figure S5A) whereas the additional promoter was detectable only after 72 h exposure (Supplementary Figure S5B). To determine the promoter strength, three transcriptional *lacZ* fusions were constructed. After 48 h growth at 37°C bright blue colonies were visible for the σ^A^-dependent p*_sr1_*-*lacZ* fusion on TY agar with X-Gal, and dark-blue colonies on 3-fold diluted TY agar (Supplementary Figure S5D), whereas colonies with the *lacZ* fusion of the additional promoter were white on TY agar and slightly blue on threefold diluted TY agar, which is in agreement with an ≈144-fold weaker TSS signal. Due to the extremely low promoter activity, no β-galactosidase activity could be measured. Consequently, the very weak promoter upstream of p*_sr1_* scarcely contributes to the amount of SR1 under sporulation conditions.

### Conservation of the SR1–*kinA* mRNA interaction among the Bacillales

In 2012, we discovered 39 SR1/SR1P homologues within the Bacillales and further analysed 9 of them (23). A new BLAST analysis detected SR1 homologues in 139 species, again only in Bacillus and Geobacillus species. In only nine *Bacillus* species, we found *kinA* homologues which have substantial sequence similarity to *B. subtilis kinA*. The other *kinA* genes encode a protein of similar size and function, but perhaps different evolutionary origin of at least the 5′ part and, therefore, are not complementary to SR1. To analyse if the SR1-*kinA* mRNA interaction is conserved in the nine species, we aligned their seven complementary SR1-*kinA* regions (Supplementary Figure S6). Region D required for the initial interaction between both molecules was identical in the nine SR1 species, and in five of nine *kinA* homologues, only one nt exchange was found in D’. Regions E’, F’ and the RBS were completely conserved in the nine *kinA* homologues, and SR1 region F carried only in two species one nt exchange. By contrast, region G/G’ which was bound last in our time course experiment (Figure 6) differed in four species significantly. From these data we conclude that the SR1-*kinA* interaction is highly conserved in these 9 *Bacillus* species.

## DISCUSSION

In 2010, it was shown that IPTG-induced KinA synthesis beyond a certain threshold can lead to entry into an irreversible sporulation process independent of nutrient availability (29). This indicates that the amount of KinA has to be tightly regulated to prevent sporulation under nutrient-rich and nonstress conditions. Here, we report on a new regulator of the *kinA* gene, the trans-encoded sRNA SR1 that shares 7 complementary regions with *kinA* mRNA designated A/A’ to G/G’. SR1 inhibits translation of *kinA* mRNA by a base-pairing interaction (Figure 4A). The effect of the *sr1* deletion could be compensated by overexpression of wild-type *sr1* but not *sr1*_mD_ carrying a 7 nt exchange in region D required for the initial interaction with *kinA* mRNA. At least in enterobacteria, most translationally inhibited mRNAs are rapidly degraded, but this is an indirect effect because they are not protected by ribosomes against endoribonucleases (rev. in 1). By contrast, SR1 does not affect the stability of *kinA* mRNA (Figure 4B) excluding both degradation as consequence of lower ribosome coverage of *kinA* mRNA and the recruitment of a 5′-3′ exoribonuclease like RNase J1 as mechanism of SR1 action. This is consistent with the dispensability of the SR1-encoded peptide SR1P (Figure 4A, B)—previously shown to impact RNase J1 activity via GapA binding (20)—for *kinA* regulation. Interestingly, SR1 only marginally influences the half-life of *ahrC* mRNA either (17,24) suggesting that it might mainly employ translational control as mechanism of action. By contrast, *B. subtilis* RoxS inhibits translation and promotes mRNA decay of *ppnKB* mRNA and *sucCD* mRNA (48) whereas it activates translation of *yflS* mRNA and, independently, prevents its degradation by RNase J1 (49). The latter is rather unusual as only a few sRNAs have been shown to directly affect target mRNA degradation, among them *E. coli* RyhB (50) and *Salmonella enterica* MicC (51).

Since the 5′ region of *kinA* mRNA that binds SR1 has neither the potential for alternative folding nor contains a Rho-dependent or -independent transcription terminator, we rule out SR1-mediated transcription termination as alternative regulatory mechanism.

Although CsrA binds both *kinA* mRNA and SR1 in the nanomolar range (Supplementary Figure S7B; 24), it neither affected the SR1-*kinA* mRNA interaction (Supplementary Figure S7C) nor induced structural changes into *kinA* mRNA *in vitro* (Supplementary Figure S7D). CsrA had also no impact on sporulation (Figure 7A) or the p*_spoIIE_*-*lacZ* transcriptional fusion (Figure 4D). This is in contrast to its role in the SR1-*ahrC* mRNA system, where it introduces slight structural changes into *ahrC* mRNA around the region required for the initial contact with SR1 (24). Likewise, Hfq did not impact sporulation although it also binds to SR1 (18). However, it cannot be excluded that a so far unidentified RNA chaperone promotes the SR1-*kinA* mRNA interaction *in vivo*.

The complementary region G’ is located upstream of the *kinA* RBS, and regions F’ to A’ immediately downstream of and within the first 32 codons of *kinA* (Figure 2). In agreement with SR1 inhibiting translation, *kinA* mRNA binding initiates at the D/D’ region 10 nt downstream of the start codon followed by a rapid protection of the *kinA* RBS and E’ located immediately upstream of D’ (Figures 2,5). Whereas the SR1-*ahrC* mRNA interaction starts at the SR1 terminator stem-loop (18), SR1 region D is located in a central single-stranded stretch corroborating that an sRNA can employ different regions to pair with its targets (rev. in 1). By contrast, sRNAs of several Gram-negative bacteria use ‘seed sequences’ in their outermost 5′ ends (51,52) and *B. subtilis* RoxS and FsrA each use the same central C-rich region for binding of all of their targets (48,49,53).

The clear effects of *sr1* deletion or overexpression on three downstream genes of the *kinA* phosphorylation cascade, the early sporulation genes *spoIIE, spoIIGA* and the late sporulation gene *cotA*, all regulated directly or indirectly by the phosphorylated transcription activator Spo0A (Figure 4C), confirm the role of SR1 in the control of the histidine kinase KinA. Furthermore, different phenotypic assays (Figure 7) demonstrated that SR1 does not only regulate sporulation initiation but impacts heat, ethanol and UV resistance as well as hydrophobicity of *B. subtilis* spores and slightly influences the spore length. Moreover, it also affects the protein composition of the outer spore coat as shown for CotA and CotB. CotA is a copper-dependent laccase required for biosynthesis of the spore pigment (54). Indeed, the less brownish colour of Δ*sr1* spores is in accordance with the lower CotA content of these spores. (Figure 7D). Actually, the ≈25% higher p*_cotA_* activity in the absence of SR1 (Figure 4C) would suggest the production of higher CotA amounts. However, CotA is apparently not efficiently incorporated into the outer spore coat as its amount is lower in Δ*sr1* spores (Figure 7D). CotB is responsible for a proper structure of the spore coat layers and the spore shape. The ≈ 30% higher hydrophobicity of Δ*sr1* spores indicates a lower crust quality, most probably due to a restricted incorporation of polysaccharides by improper coat structures (55,56).

As has been also shown recently, there is a trade-off between spore quantity and quality in *B. subtilis* (57,58). The results of our measurements of AP activity and DPA content as markers for sporulation stages II/III and IV/V, respectively, revealed that the absence of SR1 restricts the time window of spore formation to an extent that prevents the assembly of a proper coat and crust. Previously, it was found that the medium conditions during spore formation influence the spore properties, and that spores produced under nutrient-rich conditions have a higher quality than those produced under starvation (59). Indeed, the analysis of heat resistance and hydrophobicity of spores formed in the presence and absence of SR1 in rich TY medium versus poor CSE medium demonstrated that the decelerating effect of SR1 on the sporulation process is only required under starvation conditions (Figure 8C, D).

**Figure 9.**
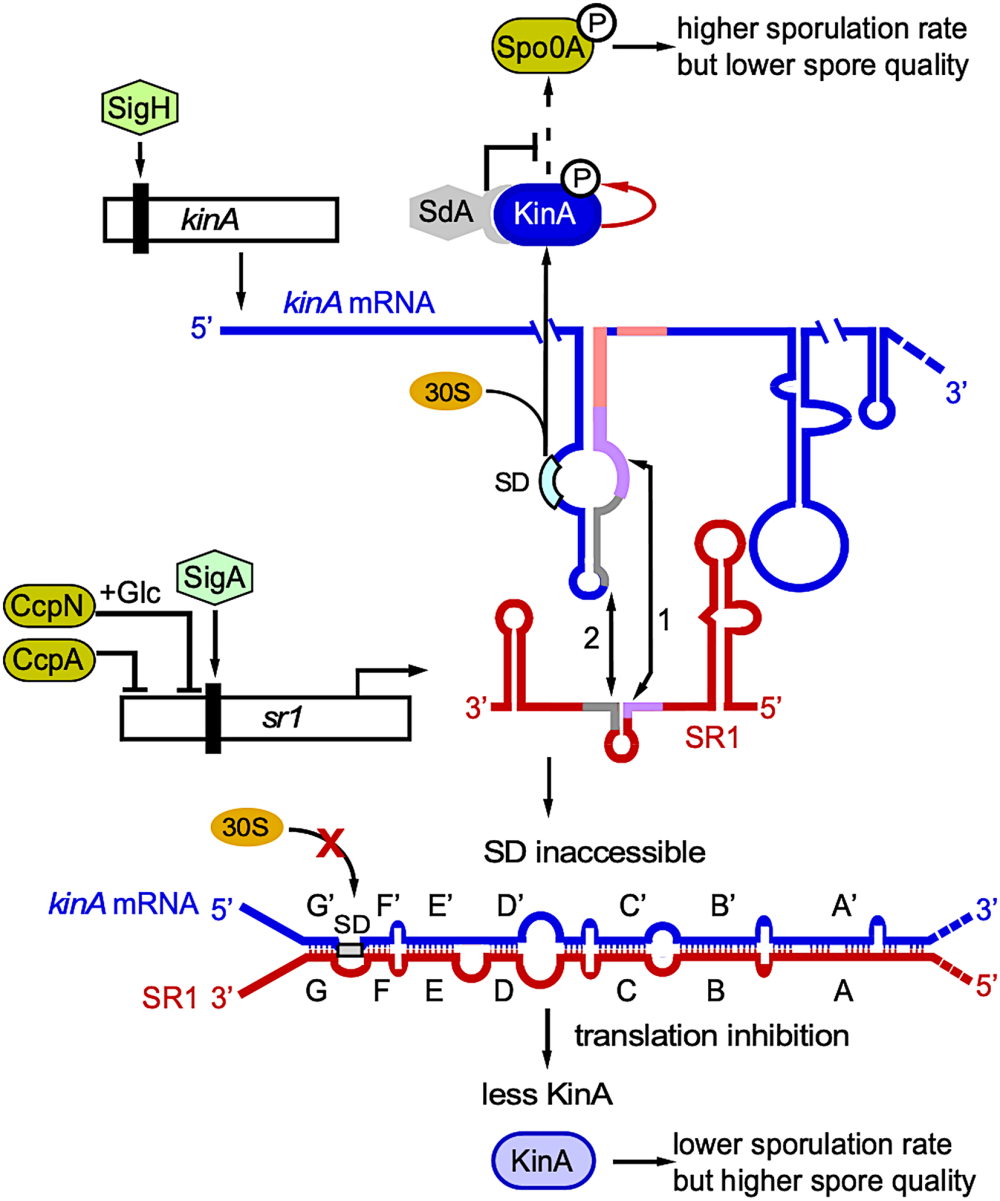
Working model on the role of SR1 in the regulation of *Bacillus subtilis* sporulation. The major histidine kinase KinA transcribed under control of σ^H^ initiates the sporulation cascade (see Figure 1). Transcription of the sRNA SR1 from p*_sr1_* is under control of σ^A^ and repressed by CcpN and CcpA during glycolysis (13–15). An additional weak promoter upstream of p*_sr1_* contributes less than 1% to the total SR1 amount (Supplementary Figure S4). SR1 interacts with *kinA* mRNA via seven complementary regions A to G. The initial interaction (1) occurs between *kinA* RNA region D’ and SR1 region D causing the protection of the *kinA* RBS (see Figures 5 and 7) followed by the E’/E (2) interactions. Thus, SR1 binding impedes the access to the ribosomal 30S subunit leading to reduced translation and, hence, lower cellular KinA levels. Consequently, less Spo0A is phosphorylated resulting in reduced sporulation but also higher spore quality. SR1 is drawn in red, *kinA* mRNA in blue. Sda (grey) directly interacts with KinA to block phosphate transfer to Spo0F, and, afterwards, to the central regulator Spo0A. Sigma factors are depicted by hexagons, transcription factors by green-brown ovals. Purple and grey lines highlight the complementary RNA regions D/D’ and E/E’ in SR1 and *kinA* mRNA.

Figure 9 presents a working model on the role of SR1 in spore formation. Why is, in addition to σ^H^ and two proteinaceous regulators, Sda and KipI, an sRNA involved in regulation of *kinA*? The bulk of SR1 is expressed under gluconeogenic conditions from the σ^A^-dependent promoter p*_sr1_* (13) whereas ≈150-fold lower SR1 amounts originating from a weak upstream promoter can be neglected (Supplementary Figure S5). The short-lived SR1 (half-life ≈ 3 min) adds an additional layer of regulation that allows to incorporate the information on the availability of various sugars (13). In the presence of sugars, i.e. the absence of SR1, higher KinA amounts accelerate the process of spore formation and maturation yielding imperfect spores with reduced stress resistance (see Figures 7,8). This agrees with a recent report on artificial sporulation and altered spore properties due to IPTG-induced *kinA* expression (60). Therefore, we hypothesize that SR1 adjusts the amount of KinA to ensure an optimal time kinetics of spore formation for the generation of high-quality spores under restricted nutrient availability.

SR1 is the first sRNA in Bacillus that was shown to regulate sporulation. Although three previous studies detected a number of sRNAs whose expression was altered upon sporulation (61–63), for none of them a target gene has been identified or their role in sporulation elucidated. Recently, the sRNA RCd1 was found to regulate sporulation in *Clostridioides difficile* (64). RCd1 represses the excision of the *skin^Cd^*element that interrupts the *sigK* gene thus preventing σ^K^ synthesis required for the late sporulation steps. *CD1234* mRNA necessary for *skin* excision and *spoIIID* mRNA encoding the transcriptional regulator of σ^E^ and σ^K^ were proposed as direct RCd1 targets *in vitro* but have not yet been confirmed *in vivo*. In contrast to *B. subtilis*, *C. difficile* sporulation is Hfq-dependent (65) but Hfq neither stabilizes RCd1 nor promotes its interaction with its target RNAs *in vitro*. Interestingly, in *Clostridium perfringens*, the sRNA VirX (which is absent in *C. difficile*) has been demonstrated to negatively control sporulation (66). In contrast to RCd1, but similar to *B. subtilis* SR1, VirX inhibits sporulation very early, likely at the Spo0A level, as *sigE* and *sigF* transcription were strongly induced in a Δ*virX* strain. Apparently, even within the genus *Clostridium*, the sporulation control is exerted by different sRNA-mediated mechanisms.

A sequence comparison of *sr1* and *kinA* homologues revealed a high conservation of complementary regions between both RNAs in nine *Bacillus* species (Supplementary Figure S6). All other Bacillus species that encode SR1 homologues have *kinA* homologues whose primary sequences differ considerably from *B. subtilis kinA* and, therefore, do not exhibit complementarity to their SR1 species. It is not excluded that their *kinA* genes are regulated by other sRNA species.

To date, we discovered two SR1 targets that are involved in different pathways, *ahrC* mRNA regulating arginine metabolism (17,18) and *kinA* mRNA governing sporulation (this report). Future research will focus on the identification and characterization of further SR1 targets and the biological role of this sRNA in the respective pathways.

## Supplementary Material

gkab747_Supplemental_File
