# Supplementary material for: A new role for SR1 from Bacillus subtilis: regulation of sporulation by inhibition of kinA translation
Source: Nucleic Acids Res. 2021 Sep 3;49(18):10589–603. doi: 10.1093/nar/gkab747 (PMC8501984; doi:10.1093/nar/gkab747)
Supplement: gkab747_Supplemental_File [file gkab747_supplemental_file.pdf]

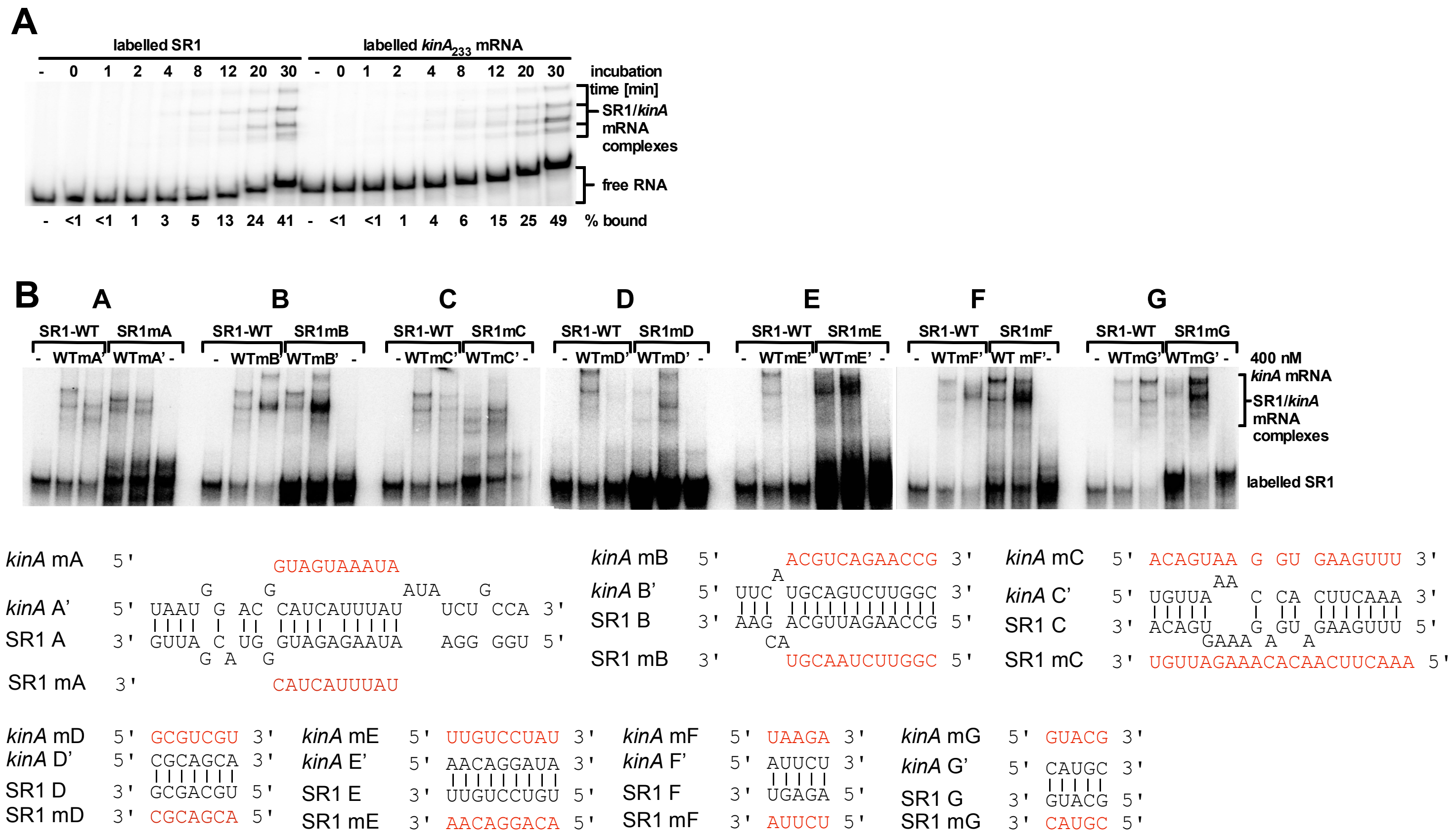

Fig. S1 ***In vitro*** **interaction between SR1 and** ***kinA*** **mRNA in EMSAs**

EMSA with 0.15 fmol ^{32}P [α-UTP]-labelled RNA and unlabelled complementary RNA. Labelled and unlabelled RNA were mixed and incubated at 37 °C in TMN buffer, followed by separation on a 6 % native PAA gel. Autoradiograms of the gels are shown. **(a)** Time course experiment with 400 nM wild-type *kinA* mRNA and SR1. **(b)** EMSA with wild-type and mutated SR1 and wild-type and mutated *kinA* mRNA species. Below, the introduced mutations in the complementary regions are shown.

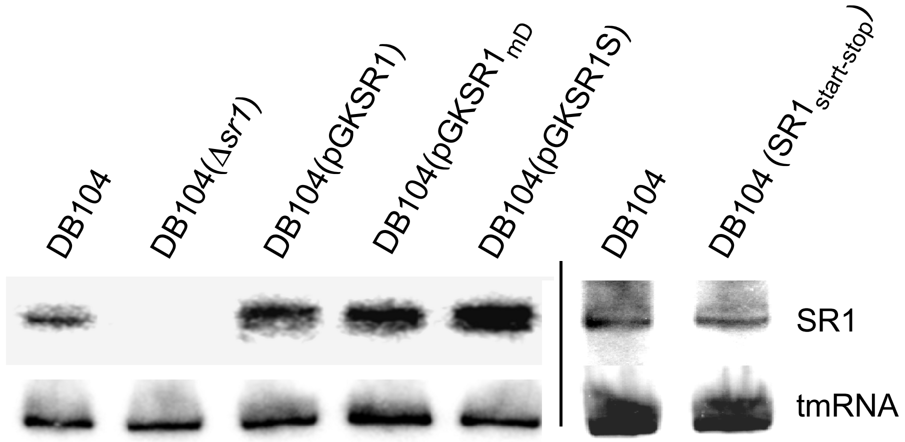

Fig. S2. Mutated SR1 species transcribed from the chromosome or from pGK vector plasmids under their native promoters are present in *B. subtilis* in comparable amounts as wild-type SR1.

Left: *B. subtilis* DB104 as well as DB104 containing pGKSR1, $pGKSR1_{mD}$ and pGKSR1S (as pGKSR1, but with spectinomycin resistance gene) were grown to OD_{600}= 5.0, total RNA prepared and subjected to Northern blotting. Right: DB104 and DB104 containing a start-to stop codon mutation in the *sr1* gene which prevents expression of the small protein SR1P) were grown to OD_{600}= 5.0, total RNA prepared and subjected to Northern blotting. ^{32}P [αUTP]-labelled riboprobes were used both for the detection of SR1 and tmRNA (loading control). The autoradiograms of the Northern blots are shown.

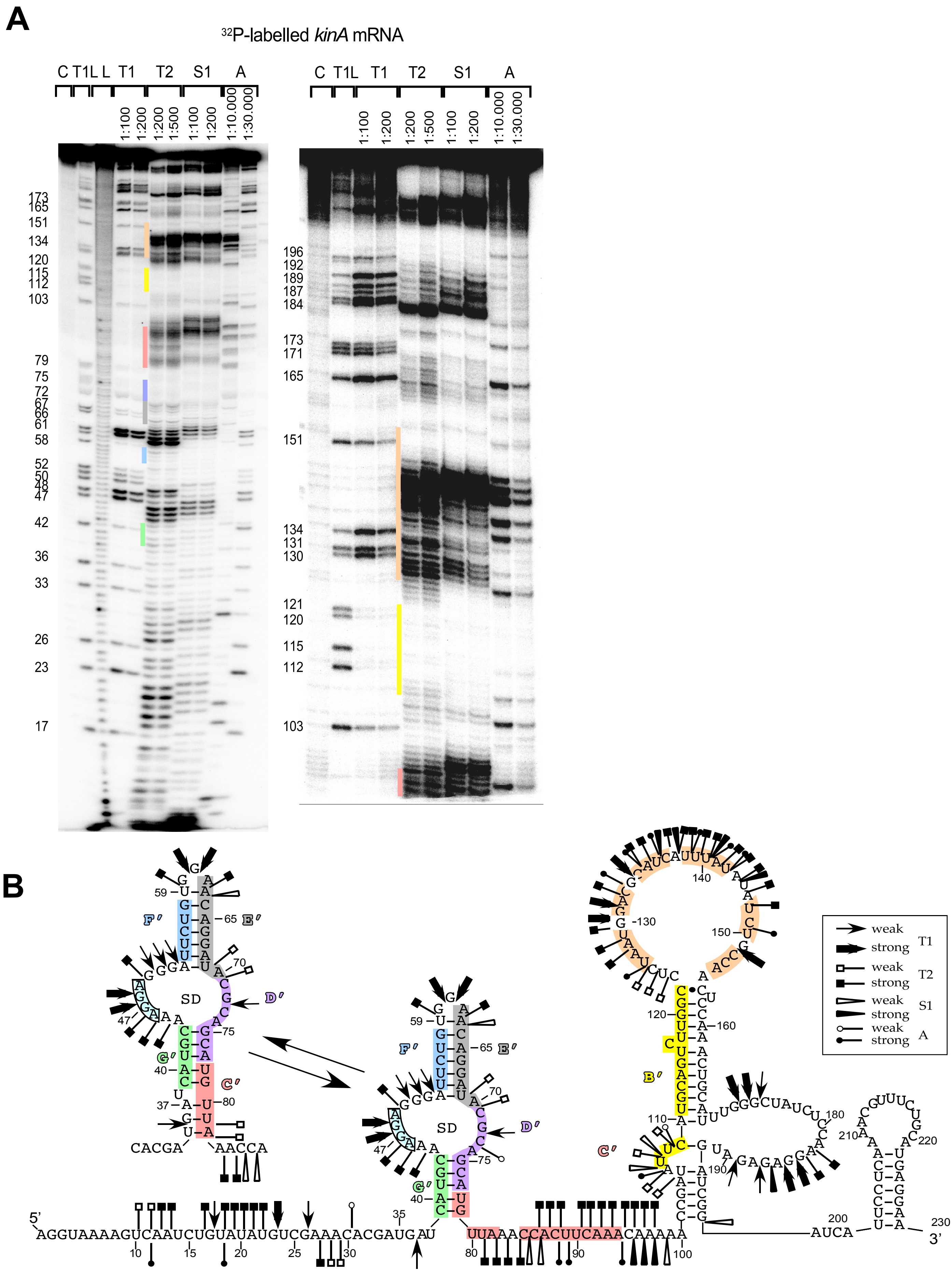

Fig. S3. Secondary structure probing of 5'-labelled *kinA*$_{233}$ mRNA

(**A**) 15 nmol of 5'-labelled *kinA* mRNA was used and RNases T1(0.1 U), T2 (0.1 U), A (0.4 ng) and nuclease S1 (2.2 U) were employed. A structure consistent with the cleavage data is depicted in (**B**). Major (dark symbols) and minor (light symbols) are indicated (see box). The regions complementary to SR1 are shown in colour code as in Fig. 2.

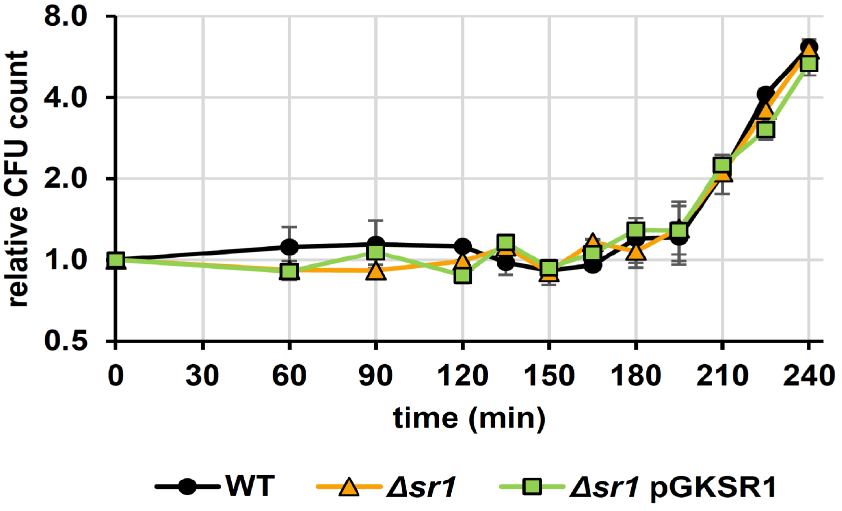

Fig. S4. Spore germination in *B. subtilis* WT, *sr1* knockout and *sr1* overexpression strains
Purified spores from wild-type strain DB104 and the isogenic *sr1* knockout and pGKSR1 overexpression strains were treated for 10 min at 70 °C, suspended in TY medium and cultivated at 37 °C under shaking for four hours. Aliquots were plated on TY agar plates at particular time points, incubated overnight at 37 °C and, subsequently, CFUs counted. The spores of all three strains displayed a lag phase of about 190 min before outgrowth indicating that the presence or absence of SR1 did not affect spore germination.

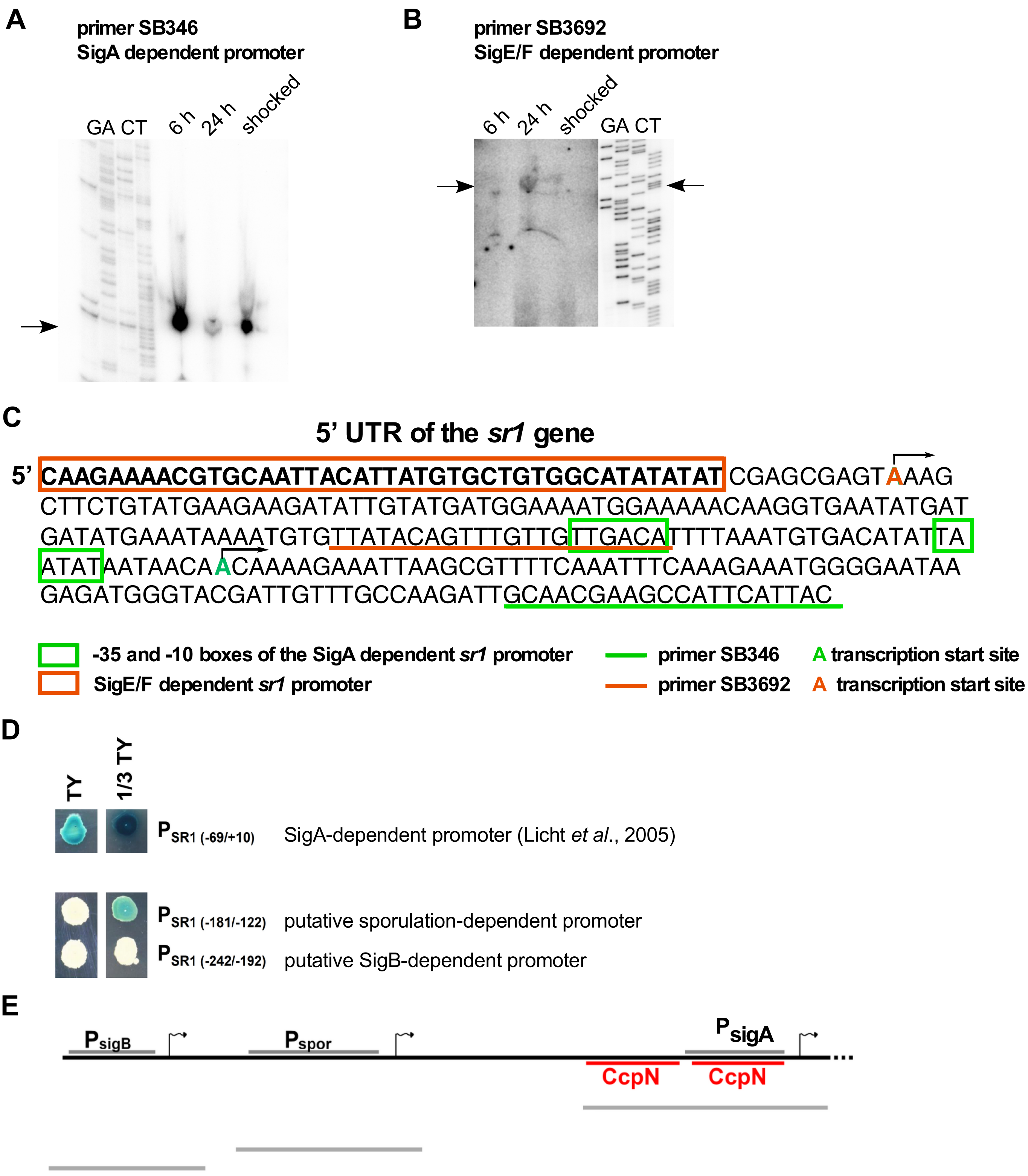

Fig. S5. Investigation of a putative sporulation-dependent promoter upstream of p_{sr1}

B. subtilis strain DB104 was grown in TY medium at 37 °C and time samples were taken after 6 h (stationary phase) and 24 h (sporulation phase). In addition, an overnight culture in TY was inoculated into fresh TY, grown for 2 h, washed with CSE minimal medium and grown for 10 min at 37 °C in CSE medium (shocked in minimal medium). From the three cultures, total RNA was prepared and used for primer extension with 5'-^{32}P-γ ATP-labelled primers SB346 (A) and SB3692 (B). For the determination of the transcription start sites, sequencing reactions with the same primers on plasmid pUC-SR1 comprising the *sr1* gene with its 5' UTR were used. Gels were exposed with PhosphorImager plates for 30 min (A) and 72 h (B), respectively. C) sequence of the 5'UTR of *sr1*. D) Investigation of four transcriptional psr1-lacZ fusions integrated into the *amyE* locus of wild-type strain DB104 grown for 48 h (sporulation) on XGal-agar plates based on TY or threefold diluted TY medium. E) Chromosomal location of the putative sporulation dependent and another putative SigB-dependent promoter. In red, the previously mapped and investigated CcpN binding sites are indicated. The grey lines below indicate the upstream regions fused the promoterless *lacZ* gene. Flexed arrows, putative transcription start sites.

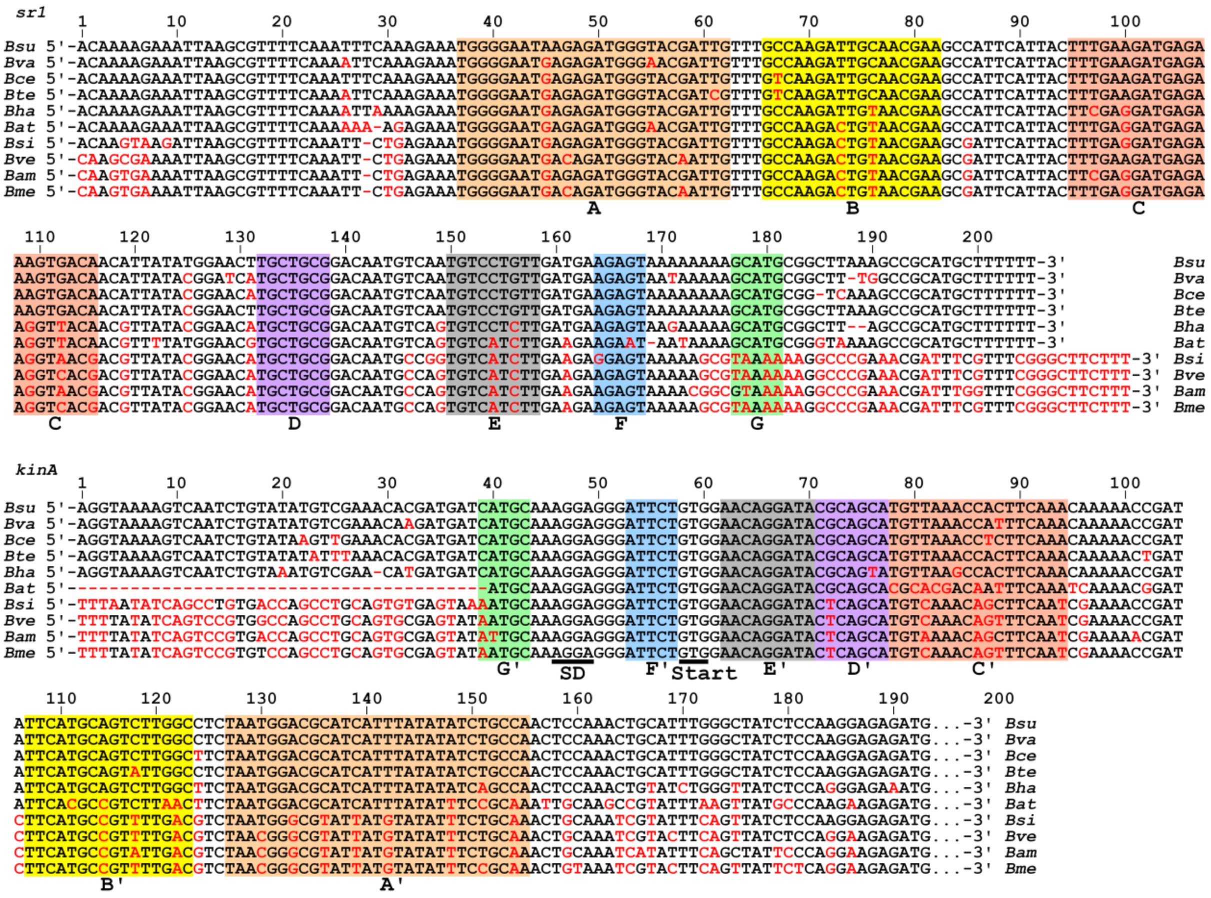

Figure S6. Sequence alignment of *sr1* and *kinA* in 9 Bacillus species

Clustal Omega alignment of *sr1* and *kinA* homologues. Nucleotides differing from the *Bacillus subtilis* 168 sequence are depicted as red. The numbering is in relation to the transcriptional start site, and complementary regions A-G and G'-A' are highlighted. The *kinA* the SD sequence and GUG start codon are underlined. *Bsu: Bacillus subtilis; Bva: Bacillus vallismortis; Bce: Bacillus cereus; Bte: Bacillus tequiliensis; Bha: Bacillus halotolerans; Bat: Bacillus atrophaeus; Bsi: Bacillus siamensis; Bve: Bacillus velezensis; Bam: Bacillus amyloliquefaciens; Bme: Bacillus methylotrophicus.*

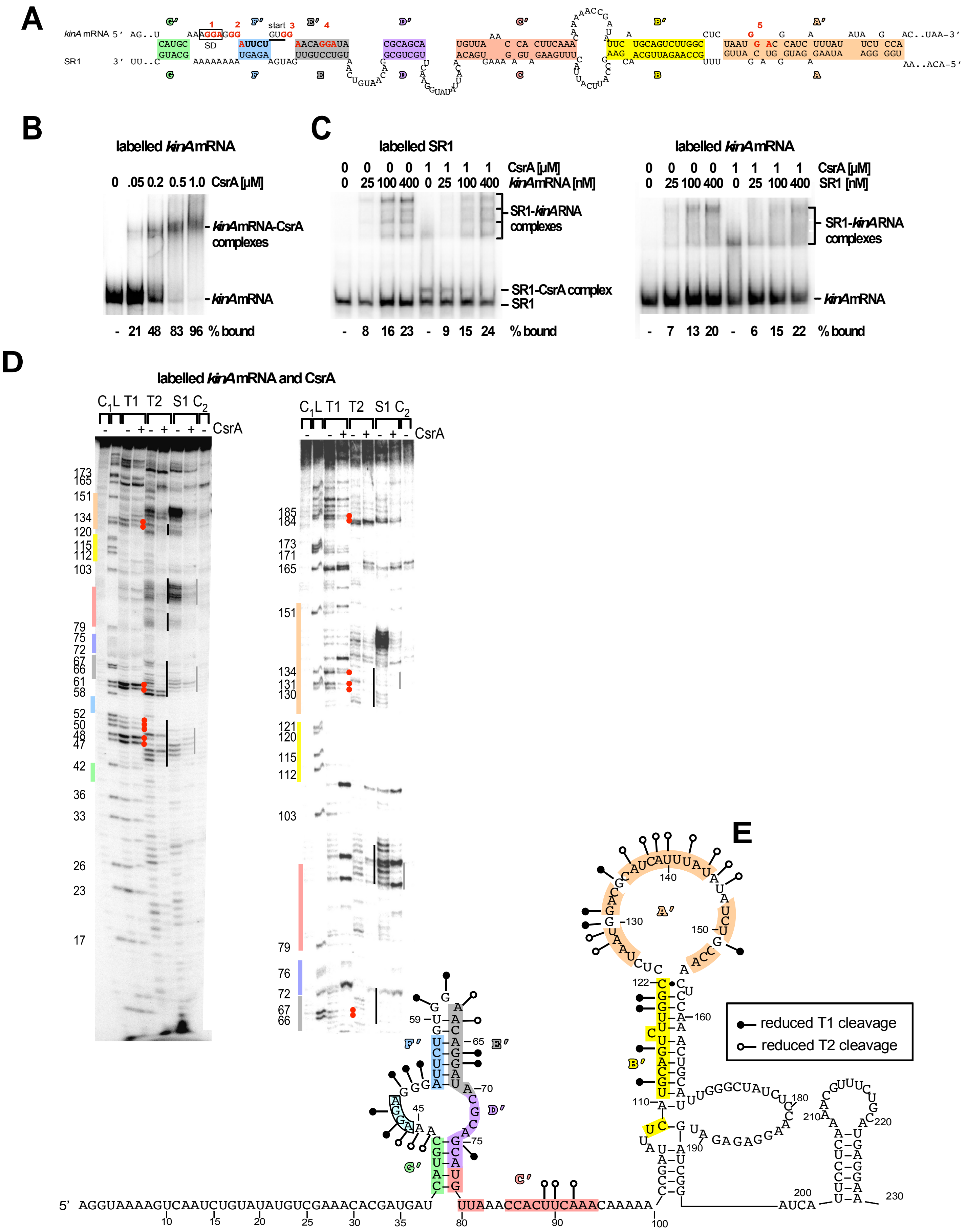

Figure S7 CsrA binds *kinA* mRNA but neither promotes SR1/*kinA* complex formation nor induces secondary structure changes into *kinA* mRNA

(a) Predicted basepairing between SR1 and $kinA_{233}$ mRNA with highlighted complementary regions. Red GGAs with numbers 1 to 4 indicate putative CsrA binding sites. **(b)** EMSA with purified internally ^{32}P-[α-UTP]-labelld $kinA_{233}$ mRNA and increasing concentration of purified CsrA. 0.15 fmol of labelled wild-type $kinA_{233}$ mRNA were incubated with CsrA at the indicated concentrations in a total volume of 10 µl (final RNA concentration 0.015 nM) for 10 min at 37 °C followed by separation on a 6 % PAA gel. **(c)** Left: Complex formation between internally labelled SR1 and unlabelled $kinA_{233}$ mRNA. Used *kinA* mRNA concentrations are indicated. 1 µM CsrA was employed if indicated and complex formation monitored in 6% PAA gels as described in Materials and Methods. The labelled RNAs (0.15 fmol/reaction) were used in at least 100-fold lower equimolar amounts than the unlabelled RNAs. **(d)** Secondary structure probing of $kinA_{233}$ mRNA in the absence and presence of CsrA. 15 nmol of 5' labelled $kinA_{233}$ mRNA and RNase T1 (0.1 U) were used. **(e)** Schematic presentation of *kinA* mRNA with the protected regions indicated as shown in the box.

Table S1: Oligonucleotides used in this study

Name	Sequence	Purpose
C767	5’ GGG TGT GAC CTC TTC GCT ATC GCC ACC	5S rRNA probe
SB317	5‘ GAA TTC AAC TTA AAA AAT AAA AGC ATG CGG CTT	Downstream primer *sr1*
SB421	5‘ GCA GGA TCC TGT TAT ATA ATT GAA ACC GTT	Upstream primer *sr1*
SB350	5’ GAA TGG ATC CGA GTA AAG CTT CTG TAT GA	Upstream primer *sr1*
SB3444	5’ GAA ATT AAT ACG ACT CC TAT AGG AGG TAA AAG TCA ATC TGT ATA TGT CGA	T7-kinA FW for IVT
SB3446	5’ TGG TCT TCC TCA TGC AGA AAC GTT	*kinA* REV for IVT
SB1	5’ GAA ATT AAT ACG ACT CAC TAT AGG CAG ATA TTT AAG TAT CTG ATT A	RNAIII FW for IVT
SB2	5’ AAG AGC CAC GAC CAG TTA A	RNAIII REV for IVT
SB3501	5’ GTA GAT GGA TCC CCA TGG TTA ATC ACT TGT TTA TTA AGA TAT TAA AAG CTA TAA TTT AAA TAG GTA AAA GTC AAT CTG TAT ATG T	pI-*kinA-lacZ* translational fusion FW
SB3465	5’ GTA GAT GAA TTC AGC TCT TGT CGT AAC AAT TTC TAC	REV for *kinA-lacZ* fusion
SB3543	5’ GAT GAA TTC TCC GGA AAT TCT TTT CAT AAA CGA	P*spoIIE-lacZ* for pMG16 FW
SB3544	5’ GAT GGA TCC TTA TAT TCG TTG CCT GTC ATT ATA	P*spoIIE-lacZ* for pMG16 REV
SB3545	5’ GAT GAA TTC TTA TAT CCT CTC ATT ATA CTT CCT	P*spoIIGA-lacZ* for pMG16 FW
SB3546	5’ GAT GGA TCC CCT TCT TGC TTC ATA AGT ATA AAG	P*spoIIGA-lacZ* for pMG16 RV
SB3547	5’ GAT GAA TTC TGA TCA AAT CCT AAA CGG CCT´GCC	P*cotA-lacZ* for pMG16 FW
SB3548	5’ GAT GGA TCC TTT CCA AAT TGG TAC TAT AGT TAA	P*cotA-lacZ* for pMG16 REV
SB3641	5’ CAT CAA CAG GAC ATT GAC ATT GTC GCG TCG TAG TTC CAT ATA ATG TTG TCA CTT T	SR1 mD upstream
SB3642	5’ AAA GTG ACA ACA TTA TAT GGA ACT ACG ACG CGA CAA TGT CAA TGT CCT GTT GAT G	SR1 mD downstream
SB625	5’ GAA ATT AAT ACG ACT CAC TAT AGG ACA AAA GAA ATTAAG CGT	FW SR1 with T7 promoter
SB303	5’ GTA ATC CTT GCA TTC CTT CG	FW SR1 NB probe (PCR)
SB309	5’ GCG TAT TGA GGC GAT GCA CC	REV SR1 NB probe (PCR)
SB3603	5’ AGC GTT TTC AAA TTT CAA AGA AAT CCC CTT ATT CTC TTG GGT ACG ATT GTT TGC CAA GAT T	SR1 delta A 2nd part FW
SB3604	5’ AAT CTT GGC AAA CAA TCG TAC CCA AGA GAA TAA GGG GAT TTC TTT GAA ATG AAA ACG CT	SR1 delta A 1st part REV
SB3605	5‘ GAA TAA GAG ATG GGT ACG ATT GTT ACG GTT CTA ACG TAC GAA GCC ATT CAT TAC TTT GAA G	SR1 delta B 2nd part FW
SB3606	5‘ CTT CAA AGT AAT GAA TGG CTT CGT ACG TTA GAA CCG TAA CAA TCG TAC CCA TCT CTT ATT C	SR1 delta B 1st part REV
SB3607	5‘ ACT TCA AAC AAA AAC CGA TAT TCA ACG TCA GAA CCG CTC TAA TGG ACG CAT CAT TTA TAT	*kinA* delta B‘ 2nd part FW
SB3608	5‘ ATA TAA ATG ATG CGT CCA TTA GAG CGG TTC TGA CGT TGA ATA TCG GTT TTT GTT TGA AGT	*kinA* delta B’ 11t part REV
SB3609	5‘ TGC AGT CTT GGC CTC TAA TGG ACG GTA GTA AAT ATA TAG ACC CAA CTC CAA ACT GCA TTT GGG CT	*kinA* delta A‘ 2nd part FW
SB3610	5‘ AGC CCA AAT GCA GTT TGG AGT TGG GTC TAT ATA TTT ACT ACC GTC CAT TAG AGG CCA AGA CTG CA	*kinA* delta A’ 11t part REV
SB3639	5‘ TCC GCA GCA AGT TCC ATA TAA TGT ACA GTC TTT GTG TTG AAG TTT GTA ATG AAT GGC TTC GTT GCA ATC	SR1 delta C 2nd part FW
SB3640	5‘ GAT TGC AAC GAA GCC ATT CAT TAC AAA CTT CAA CAC AAA GAC TGT ACA TTA TAT GGA ACT TGC TGC GGA	SR1 delta C1 1t part REV
SB3641	5‘ CAT CAA CAG GAC ATT GAC ATT GTC GCG TCG TAG TTC CAT ATA ATG TTG TCA CTT T	SR1 delta D 2nd part FW
SB3642	5‘ AAA GTG ACA ACA TTA TAT GGA ACT ACG ACG CGA CAA TGT CAA TGT CCT GTT GAT G	SR1 delta D1 1t part REV
SB3643	5‘ GCA TGC TTT TTT TTA CTC TTC ATC TTG TCC TGT TTG ACA TTG TCC GCA GCA AGT TCC	SR1 delta E 1st part FW
SB3644	5‘ GGA ACT TGC TGC GGA CAA TGT CAA ACA GGA CAA GAT GAA GAG TAA AAA AAA GCA TGC	SR1 delta E 2nd part REV
SB3645	5‘ GCT TTA AGC CGC ATG CTT TTT TTT TGA GAT CAT CAA CAG GAC ATT GAC ATT GT	SR1 delta F 1st part REV
SB3646	5‘ ACA ATG TCA ATG TCC TGT TGA TGA TCT CAA AAA AAA AGC ATG CGG CTT AAA GC	SR1 delta F 2nd part FW
SB3647	5‘ AAG CAT GCG GCT TTA AGC CGG TAC GTT TTT TTT ACT CTT CAT CAA CAG G	SR1 delta G 1st part REV
SB3648	5‘ CCT GTT GAT GAA GAG TAA AAA AAA CGT ACC GGC TTA AAG CCG CAT GCT T	SR1 delta G 2nd part FW
SB3649	5‘ AGA CTG CAT GAA TAT CGG TTT TTG AAA CTT CAC CTT ATT GTT GCT GCG TAT CCT GTT CCA CAG AA	*kinA* delta C‘ - 1st part REV
SB3650	5‘ TTC TGT GGA ACA GGA TAC GCA GCA ACA ATA AGG TGA AGT TTC AAA AAC CGA TAT TCA TGC AGT CT	*kinA* delta C‘ - 2nd part FW

SB3651	5' GTT TTT GTT TGAA GTG GTT TAA CAA CGA CGC TAT CCT GTT CCAC AGA ATC CCT CC	*kinA* delta D' - 1st part REV
SB3652	5' GGA GGG ATT CTGT GGA ACA GGA TAG CGT CGT TGT TAA ACC ACT TCA AAC AAA AAC	*kinA* delta D' - 2nd part FW
SB3653	5' TTT GAA GTG GTT TAA CAT GCT GCG ATA GGA CAA CCA CAG AAT CCC TCC TTT GCA TGA	*kinA* delta E' – 1st part REV
SB3654	5' TCA TGC AAA GGA GGG ATT CTG TGG TTG TCC TAT CGC AGC ATG TTA AAC CAC TTC AAA	*kinA* delta E' - 2nd part FW
SB3655	5' AAC ATG CTG CGT ATC CTG TTC CAC TCT TAC CCT CCT TTG CAT GAT CAT CGT GT	*kinA* delta F' – 1st part REV
SB3656	5' ACA CGA TGA TCA TGC AAA GGA GGG TAA GAG TGG AAC AGG ATA CGC AGC ATG TT	*kinA* delta F' - 2nd part FW
SB3657	5' CCT GTT CCA CAG AAT CCC TCC TTT CGT ACA TCA TCG TGT TTC GAC ATA T	*kinA* delta G' – 1st part REV
SB3658	5' ATA TGT CGA AAC ACG ATG ATG TAC GAA AGG AGG GAT TCT GTG GAA CAG G	*kinA* delta G' - 2nd part FW
SB3550	5' GAT GAA TTC AGT TCA AGA AAA CGT GCA ATT ACAT	FW – p_{spor}-promoter for pMG16 (-181 -> x)
SB3552	5' GAT GGA TCC AGA AGC TTT ACT CGA TAT ATA TGC	REV – p_{spor}-promoter for pMG16 (x -> -122)
SB3549	5' GAT GAA TTC TAG TTT ATC ACG AGA GAC GGC TTA	FW – S533-promoter for pMG16 (-331 -> x)
SB3553	5' GAT GGA TCC ACA ACA AAC TGT ATA ACA CAT TTT	REV – S533-promoter for pMG16
SB3700	5' AAT TCG ATA TGA AAT AAA ATG TGT TAT ACA GTT TGT TGT TGA CAT TTT AAA TGT GAC ATA TTA ATA TAA TAA CAA CAA AAG AAA G	FW – SR1 promoter with CcpN-sites (-69->+10)
SB3701	5' GAT CCT TTC TTT TGT TGT TAT TATA TTA ATA TGT CAC ATT TAA AAT GTC AAC AAC AAA CTG TAT AAC ACA TTT TAT TTC ATA TCG	REV– SR1 promoter with CcpN-sites (-69->+10)
SB3704	5' AAT TCA TCA ATC GTT TAA AAT AGC AAC CTG GGG TAT ATT GAA TTT TTT GCT GCA TTG	FW – SigB-promoter upstream SR1 (-242->-192)
SB3705	5' GAT CCA ATG CAG CAA AAA ATT CAA TAT ACC CCA GGT TGC TAT TTT AAA CGA TTG ATG	REV – SigB-promoter upstream SR1 (-242->-192)
SB346	5' GTAATGAATGGCTTCGTTGC	SR1 primer extension 1
SB3692	5' TGTCAACAACAAACTGTATAA	SR1 primer extension 2
SB3055	5' CCT ATA GAA GCG GAT TTG TCT TGG CGG CGT CCT ACT CTC A	RT primer 5S rRNA
SB3766	5' CAA GAA CAT CTG TAT TCG AAG GAA TGA TCC GAT CAT CTC TCC	RT primer *kinA* mRNA
SB3765	5' ATT CAT GCA GTC TTG GCC TC	qPCR forward *kinA* mRNA
SB3054	5' CAA GAA CAT CTG TAT TCG AAG	qPCR reverse *kinA* mRNA
SB3057	5' AGC GAA GAG GTC ACA CCC GT	qPCR forward 5S rRNA
SB3058	5' AGC GAA GAG GTC ACA CCC GT	qPCR reverse 5S rRNA
SB2938	5' TATATTTATGTTACAGTAATA	Chloramphenicol cassette
SB2939	5' AAC TAA CGG GGC AGG TTA GTG	Chloramphenicol cassette
SB3769	5' AAT GGG GAA TAA GAG **TAA** GGT ACG ATT GTT TGC	FW *sr1 start-to stop*
SB3770	5' GCA AAC AAT CGT ACC **TTA** CTC TTA TTC CCC ATT	Rev *sr1 start-to stop*
SB3771	5' TAT TAC TGT AAC ATA AAT ATA TAA AGC TTC TGT ATG AAG AAG A	Outer primer1 *sr1 start-stop*
SB3772	5' GAA GGC CTT GTA TCT AAA GAC	Outer primer2 *sr1 start-stop*
SB3773	5'CAC TAA CCT GCC CCG TTA GTT TGC CAC AGC ACA TAA TGT AAT	*sr1 start-stop* back cassette
SB3774	5' ACAGGCGAAAGAAGAAGGTCT	*sr1 start-stop* back cassette

FW, forward primer; IVT, *in vitro* transcription; REV, reverse primer; NB, Northern blot

Table S2: Bacterial strains used in this study

Strain	Genotype	Reference
E. coli TG1	F' *[traD36 proAB lacIqZ ΔM15] supE thi-1 Δ(lac-proAB) Δ(mcrB-hsdSM)5(rK⁻ mK⁻)*	Rowitch *et al*., 1988
B. subtilis DB104	*His, nprR,2 nprE18, ΔaprA3*	Kawamura Doi, 1984
DB104(*amyE::kinA-lacZ*)	DB104 with *kinA-lacZ* translational fusion, Km^R	This study
DB104(amyE::*kinAmD-lacZ*)	DB104 with *kinAmD-lacZ* translational fusion, Km^R	This study
B. subtilis BK13990	*B. subtilis* 168(*ΔkinA::erm trpC2*), *kinA* knockout	Ohio strain collection
DB104(*ΔkinA::erm*)	DB104 lacking the *kinA* gene, Em^R	This study
DB104 *(kinA::erm, sr1::phleo, amyE::kinA-lacZ)*	DB104 lacking *kinA* and *sr1*; *kinA-lacZ*, Em^R, Pm^R, Km^R	This study
DB104 *(kinA::erm, sr1::phleo, amyE::kinAmD-lacZ)*	DB104 lacking *kinA* and *sr1*; *kinAmD-lacZ*, Em^R, Pm^R, Km^R	This study
DB104 (*amyE*::p_{spoIIE}-*lacZ*)	DB104 with transcriptional p_{spoIIE}-*lacZ* fusion, $Spec^R$	This study
DB104 (*amyE*::$p_{spoIIGA}$-*lacZ*)	DB104 with transcriptional $p_{spoIIGA}$-*lacZ* fusion, $Spec^R$	This study
DB104 (*amyE*::p_{cotA}-*lacZ*)	DB104 with transcriptional $p_{cotA\text{-}lacZ}$ fusion, $Spec^R$	This study
DB104 ($sr1_{start\text{-}to\ stop}$)	DB104 with start-to stop codon mutation in *sr1p*, Cm^R	This study

Ap^R, ampicillin resistance, Em^R, erythromycin resistance; Km^R, kanamycin resistance; Pm^R, phleomycin resistance; $Spec^R$, spectinomycin resistance.

Table S3: Plasmids used in this study

Plasmid	Description	Reference
pGAB1	Vector for integration of translational *lacZ* fusions into the *amyE* locus, Amp^R, Km^R	Müller *et al.,* 2019
pMG16	Vector for integration of transcriptional *lacZ* fusions into *amyE* locus, Ap^R, $Spec^R$	Müller *et al*., 2016
pGKSR1	Vector for replication in *E. coli* and *B. subtilis*, comprising *sr1* gene with p_{sr1}, Em^R	Licht *et al*., 2005
pGKSR1$_{mD}$	As pGKSR1, but with 7 bp exchange in SR1 region D	This study
pGK15	Vector for replication in *E. coli* and *B. subtilis*, $Spec^R$	M. Gimpel, unpublished
pGKSR1S	pGK15 with *sr1* gene from pGKSR1, $Spec^R$	This study
pUCSR1	pUC19 with *sr1* gene comprising 250 bp upstream of the *sr1* promoter, Ap^R	This study

Ap^R, ampicillin resistance, Em^R, erythromycin resistance; Km^R, kanamycin resistance; $Spec^R$, spectinomycin resistance.

References for Supplementary Material

1. Rowitch DH, Hunter GJ, Perham RN (1988) Variable electrostatic interaction between DNA and coat protein in filamentous bacteriophage assembly. *J. Mol. Biol.* **204**, 663-674.
2. Kawamura,F. and Doi,R.H. (1984) Construction of a *Bacillus subtilis* double mutant deficient in extracellular alkaline and neutral proteases. *J. Bacteriol.,* **160**, 442-444.
